# The Kynurenine Pathway and Indole Pathway in Tryptophan Metabolism Influence Tumor Progression

**DOI:** 10.1002/cam4.70703

**Published:** 2025-03-18

**Authors:** Zhanhui Lu, Chengcheng Zhang, Jia Zhang, Wan Su, Guoying Wang, Zhongqi Wang

**Affiliations:** ^1^ Department of Medical Oncology, Longhua Hospital Shanghai University of Traditional Chinese Medicine Shanghai China; ^2^ Shanghai University of Traditional Chinese Medicine Shanghai China; ^3^ Cancer Institute, Longhua Hospital Shanghai University of Traditional Chinese Medicine Shanghai China; ^4^ Provincial Hospital Affiliated to Shandong First Medical University Jinan Shandong China; ^5^ Department of Critical Care Medicine The Second People's Hospital of Dongying Dongying Shandong China

**Keywords:** indole, kynurenine, mechanism, tryptophan metabolism, tumor immunology

## Abstract

Tryptophan (Trp), an essential amino acid, is solely acquired through dietary intake. It is vital for protein biosynthesis and acts as a precursor for numerous key bioactive compounds. The Kynurenine Pathway and the Indole Pathway are the main metabolic routes and are extensively involved in the occurrence and progression of diseases in the digestive, nervous, and urinary systems. In the Kynurenine Pathway, enzymes crucial to tryptophan metabolism, indoleamine‐2,3‐dioxygenase 1 (IDO1), IDO2, and Trp‐2,3‐dioxygenase (TDO), trigger tumor immune resistance within the tumor microenvironment and nearby lymph nodes by depleting Trp or by activating the Aromatic Hydrocarbon Receptor (AhR) through its metabolites. Furthermore, IDO1 can influence immune responses via non‐enzymatic pathways. The Kynurenine Pathway exerts its effects on tumor growth through various mechanisms, including NAD+ regulation, angiogenesis promotion, tumor metastasis enhancement, and the inhibition of tumor ferroptosis. In the Indole Pathway, indole and its related metabolites are involved in gastrointestinal homeostasis, tumor immunity, and drug resistance. The gut microbiota related to indole metabolism plays a critical role in determining the effectiveness of tumor treatment strategies and can influence the efficacy of immunochemotherapy. It is worth noting that there are conflicting effects of the Kynurenine Pathway and the Indole Pathway on the same tumor phenotype. For example, different tryptophan metabolites affect the cell cycle differently, and indole metabolism has inconsistent protective effects on tumors in different regions. These differences may hold potential for enhancing therapeutic efficacy.

## 1 Introduction

Tryptophan plays an irreplaceable role in the health and diseases of humans as an essential amino acid. It is involved in the synthesis of various important biochemical compounds. As research on the tryptophan pathway has intensified, it has been found to have important roles in neurological, digestive, aging, immune, infection, and tumor‐related diseases [1].

In the human body, although the majority of tryptophan (Trp) binds to albumin, 5%–10% remains as free tryptophan that does not bind to albumin. Only when tryptophan dissociates from albumin can it be metabolized by enzymes, impacting physiological processes. Approximately 5% of these free tryptophans are utilized in protein and serotonin synthesis [2], while 95% are converted through the Kynurenine Pathway to produce various bioactive compounds [3]. Free tryptophan levels in the serum are primarily influenced by dietary intake and metabolic pathways. Currently, the metabolic pathways of tryptophan are summarized as proceeding mainly through three routes. The first is the oxidation of Trp via the Kynurenine Pathway (KP), with approximately 95% of Trp being metabolized through this route. This pathway is associated with the development of depression, functional gastrointestinal disorders, cardiovascular diseases, neurodegenerative diseases, immune disorders, and tumors [4]. The second pathway is the hydroxylation of Trp through the Serotonin Pathway (SP), which plays a critical role in the gut‐brain axis and is linked to various neuropsychiatric disorders [5]; serotonin has been shown to possess anti‐inflammatory properties, providing neuroprotective effects. However, in various cancers, it may promote cancer progression by enhancing angiogenesis and modulating the immune system [6]. The third pathway involves the transamination of tryptophan, which contributes to tumor progression through the production of indole‐3‐propionic acid (IPA) and kynurenic acid (KYNA) [7, 8]. Additionally, dietary tryptophan, besides being absorbed by the gut, can be metabolized through the Indole Pathway (IP) by gut microbiota, which is related to neuropsychiatric disorders, inflammatory bowel disease (IBD), colorectal cancer, metabolic diseases, and immune system function [9, 10, 11, 12].

This paper focuses on the role of tryptophan metabolism in malignant tumors, combs through the major pathways of tryptophan metabolism, summarizes, and discusses the roles played by the Kynurenine Pathway and Indole pathway in tumors.

### 1.1 Kynurenine Pathway

Tryptophan (Trp) is oxidized to *N*‐formylkynurenine (NFK) under the action of the enzymes indoleamine‐2,3‐dioxygenase 1 (IDO1), IDO2, and Trp‐2,3‐dioxygenase (TDO). NFK is then hydrolyzed to kynurenine (Kyn) by the enzyme N′‐formylkynurenine formamidase (FAMID). From this point, Kyn can follow three different pathways:

1. Deamination by Kyn aminotransferases (KAT I–IV) forms kynurenic acid (KYNA).
2. Hydrolysis by kynureninase (KYNU) to produce Anthranilic acid (AA).
3. Monooxygenation by Kyn monooxygenase (KMO) generates 3‐hydroxykynurenine (3‐HK).

**FIGURE 1:**
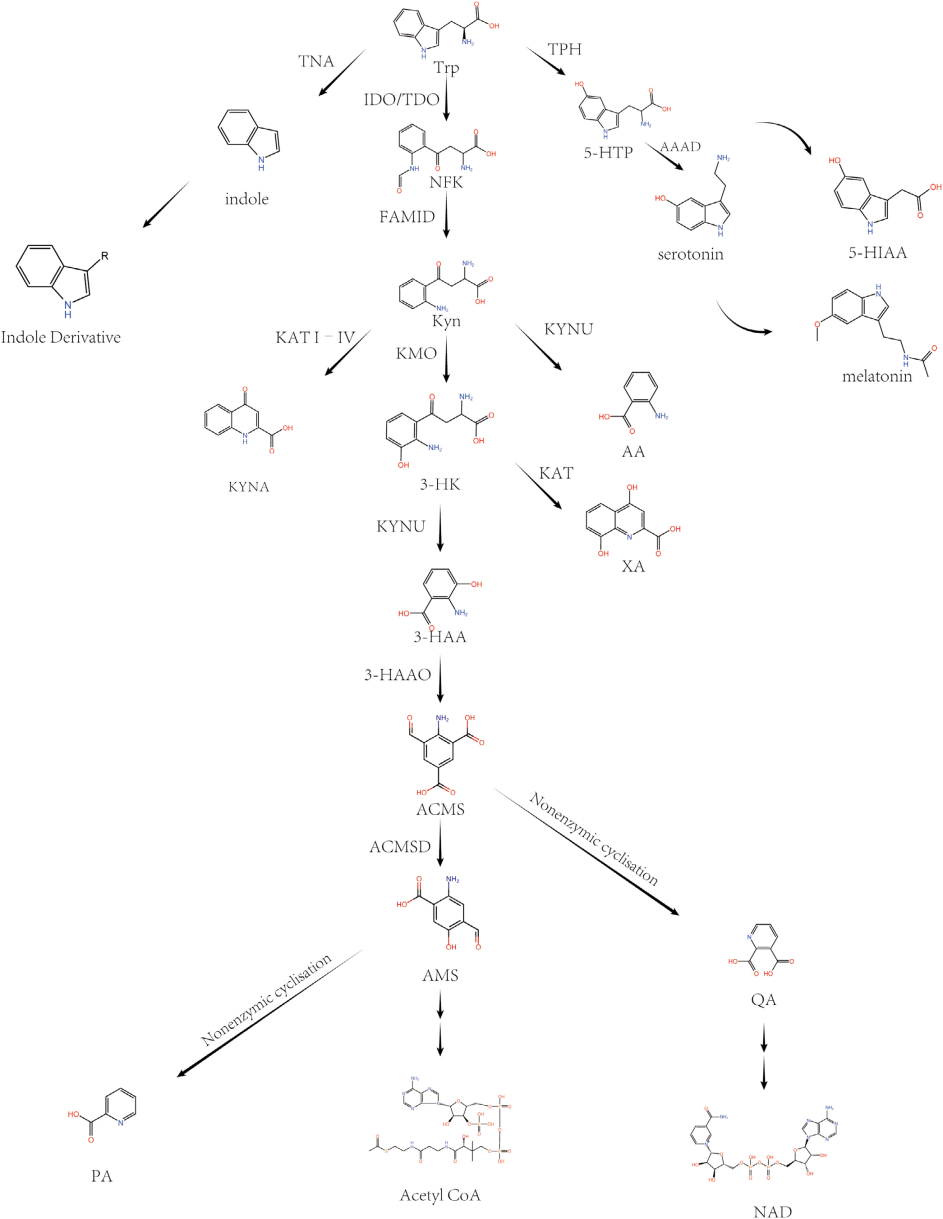
Tryptophan metabolism diagram. 3‐HAA, 3‐hydroxyanthranilic acid; 3‐HAAO, 3‐HAA 3,4‐dioxygenase; 3‐HK, 3‐hydroxykynurenine; 5‐HIAA, 5‐hydroxyindoleacetic acid; 5‐HT, serotonin; 5‐HTP, 5‐hydroxytryptophan; AA, anthranilic acid; KMO, Kyn monooxygenase; AAAD, aromatic l‐amino acid decarboxylase; ACMS, 2‐amino‐3‐carboxymuconic acid‐6‐semialdehyde; ACMSD, ACMS decarboxylase; AMS, 2‐amino‐3‐muconic acid‐6‐semialdehyde; FAMID, *N*′‐formylkynurenine formamidase; IDO, indoleamine‐2,3‐dioxygenase; KAT, Kyn aminotransferases; Kyn, kynurenine; KYNA, kynurenic acid; KYNU, kynureninase; PA, picolinic acid; QA, quinolinic acid; TDO, tryptophan‐2,3‐dioxygenase; TNA, tryptophanase; TPH, tryptophan hydroxylase; Trp, tryptophan; XA, xanthurenic acid.

Downstream of this pathway, 3‐HK can be further metabolized through two routes:

1. It can be converted to 3‐hydroxyanthranilic acid (3‐HAA) by KYNU or to xanthurenic acid (XA) by KAT through deamination.
2. 3‐HAA can then be processed by 3‐HAA 3,4‐dioxygenase (3‐HAAO) to form 2‐amino‐3‐carboxymuconic acid‐6‐semialdehyde (ACMS), which can non‐enzymatically cyclize into quinolinic acid (QA). Alternatively, ACMS can be converted to 2‐amino‐3‐muconic acid‐6‐semialdehyde (AMS) by ACMS decarboxylase (ACMSD, also known as picolinate carboxylase), and then non‐enzymatically cyclizes into picolinic acid (PA). In cases where AMS levels are insufficient, the pathway may favor the production of acetyl CoA. QA, through multiple metabolic steps, can ultimately be converted into NAD+ (Figure 1).

### 1.2 Serotonin Pathway

Tryptophan is primarily metabolized into serotonin (5‐HT) in two locations: the gastrointestinal tract (90%) and the central nervous system (10%) [13]. Because of the presence of the blood–brain barrier (BBB), 5‐HT synthesized in the periphery cannot act on the central nervous system. Under the action of tryptophan hydroxylase 1/2 (TPH1/2), Trp is hydroxylated to 5‐hydroxytryptophan (5‐HTP). TPH1 is mainly distributed in the enterochromaffin cells of the intestine, and TPH2 is found in the central nervous system and enteric nervous system, where it also serves as the key rate‐limiting step. 5‐HTP is converted into 5‐hydroxytryptamine (5‐HT) by aromatic L‐amino acid decarboxylase (AAAD). After being secreted by intestinal cells into the lamina propria, most peripheral 5‐HT is taken up and stored by platelets in the capillaries, and then exerts its effects throughout the body. In the central nervous system, 5‐HT is synthesized and stored in presynaptic neurons for neurotransmission or further transformed into melatonin. 5‐HT is oxidized by monoamine oxidase (MAO) into 5‐hydroxyindoleacetic acid (5‐HIAA), and then excreted from the body through urine.

### 1.3 The Transamination Pathway

Tryptophan (Trp) undergoes conversion to IPA through an unstable intermediate facilitated by Trp transaminase [14]. It is hypothesized that in the KP, Kyn forms KUNA with transaminase involvement, yet prior studies revealed that IPA might also form KA through an unstable kynurenine intermediate in the presence of reactive oxygen species (ROS) [7]. Additionally, reports from 30 years ago identified that KynA can be generated via I3P [15, 16], and recent studies have shown that interleukin‐4‐induced‐1 (IL4I1) catalyzes Trp to form I3P, with IL4I1‐derived H_2_O_2_ promoting the conversion of I3P to KYNA [8].

### 1.4 Indole Pathway and Gut Microbiota

**FIGURE 2:**
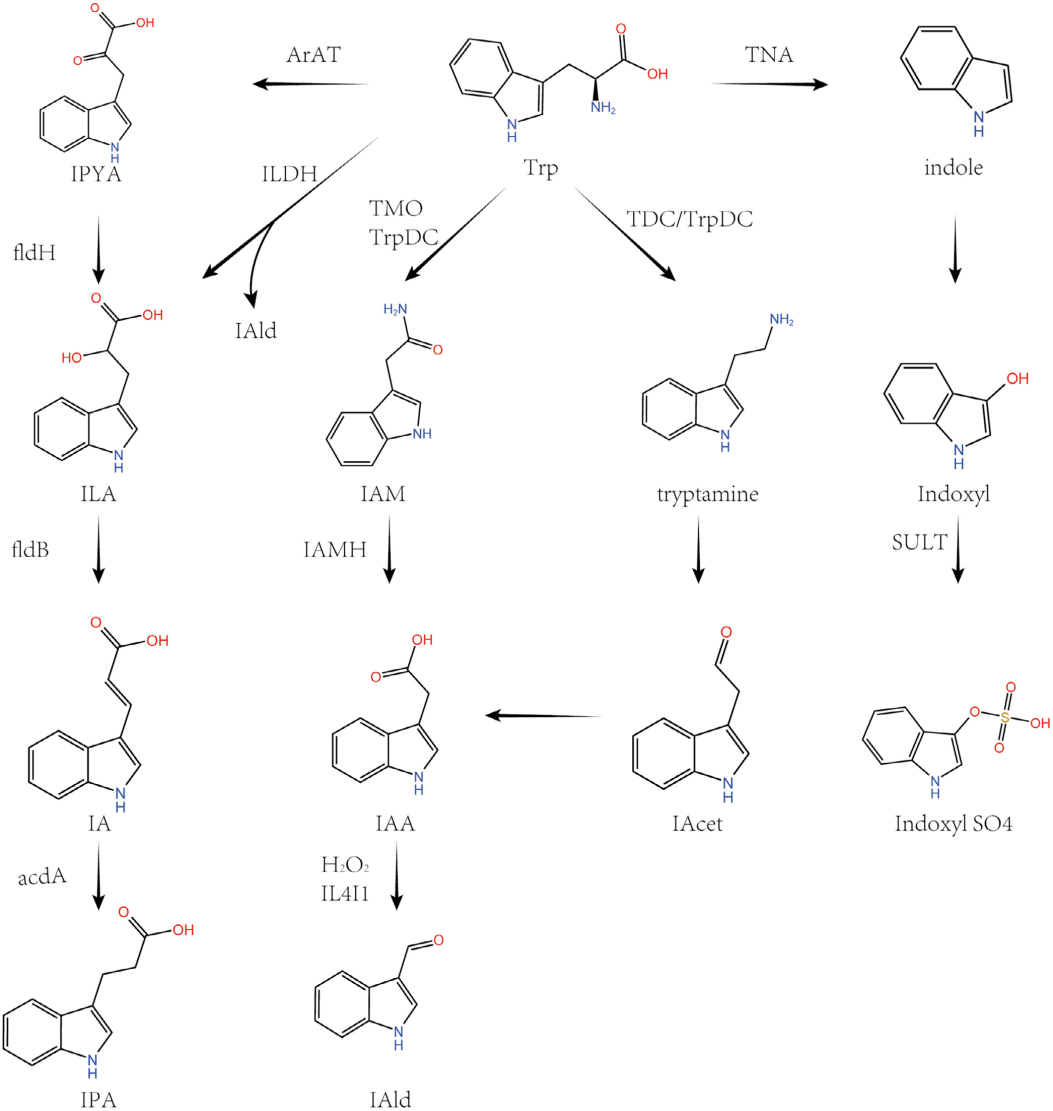
Indole pathway metabolism diagram. acdA, acyl‐CoA dehydrogenase; ArAT, aromatic amino acid aminotransferase; fldB, phenyllactate dehydratase; fldH, phenyllactate dehydrogenase; IAA, indol‐3‐ylacetic acid; IAcet, indole acetaldehyde; IAld, indole aldehyde; IAM, indole‐3‐acetamide; IAMH, indole‐3‐acetamide hydrolase; ILA, indole‐3‐lactic acid; ILDH, indolelactate dehydrogenase; IPA, indole‐3‐propionic acid; IPYA, indole‐3‐pyruvate; LA, indole‐3‐acetamide; SULT, sulphotransferase; TMO, tryptophan‐2‐monooxygenase; TNA, tryptophanase; Trp, tryptophan; TrpDC, tryptophan decarboxylase.

Most tryptophan is absorbed in the proximal intestine, while the remainder is metabolized by microbes in the distal intestine. There are three main pathways of its metabolism (Figure 2):

1. Tryptophanase (TNA) in *Escherichia coli* and *Lactobacillus* species converts tryptophan (Trp) into indole [17, 18].
2. IPA metabolism: Aromatic amino acid aminotransferase (ArAT) is a key phylogenetically conserved enzyme found in many bacteria, including *Lactobacillus* species, which dominate in the gut of mice [19].
  Aminotransferase in *Clostridium sporogenes* ( *C. sporogenes* ) converts tryptophan (Trp) into Indole‐3‐Pyruvate (IPYA), which is then successively transformed into Indole‐3‐Lactic Acid (ILA), indoleacrylic acid (IA), and Indole‐3‐Propionic Acid (IPA) by phenyllactate dehydratase. Similar metabolic processes are found in bacteria such as *Clostridium botulinum* , three strains of *Clostridium cadaveris* ( *Clostridium cadaveris* CC88A, CC44 001G, and CC40 001C), and anaerobic *Peptostreptococcus* ( *Peptostreptococcus anaerobius* CC14N), including *P. russellii* , *P. anaerobius* , and *P. stomatis* [20].
  Additionally, in *Lactobacillus*, ArAT and indolelactate dehydrogenase (ILDH) collaborate to convert tryptophan into indole aldehyde (IAld) and ILA [21].
3. Tryptamine metabolism: Experiments by Williams confirmed that tyrosine decarboxylase (TDC) in *Clostridium sporogenes* and tryptophan decarboxylase (TrpDC) from *Ruminococcus gnavus* can decarboxylate tryptophan into tryptamine [22]. Tryptamine is oxidized and deaminated to form indole acetaldehyde (IAcet), which is further oxidized to indol‐3‐ylacetic acid (IAA). IAA can be oxidized to IAld, a process that may be driven by hydrogen peroxide (H_2_O_2_) generated in the tryptophan metabolic pathway mediated by IL4I1 [23].
4. Metabolism of indole‐3‐acetamide (IAM): Certain intestinal microbiota (e.g., *Lactiplantibacillus plantarum*) oxidize tryptophan into indole‐3‐acetamide (IAM) under the action of tryptophan‐2‐monooxygenase (TMO) and tryptophan decarboxylase, which is then converted to indole‐3‐acetic acid (IAA) by indole‐3‐acetamide hydrolase [18, 24].

Compared to germ‐free rats, Wikoff et al. found that conventional mice have specific tryptophan metabolites derived from the gut microbiota, including indolepropionic acid and indoxyl sulfate. Plasma serotonin levels in conventional mice were 2.8 times higher than in germ‐free mice, fecal tryptamine levels increased by 2‐fold, and serum tryptophan concentrations decreased [25, 26]. These findings highlight the critical role of gut microbiota in indole metabolism for the host. The following will discuss the roles of these metabolites in cancer.

## 2 The Function of Kynurenine Pathway in Cancer

### 2.1 Inhibition of Local Immune Response

**FIGURE 3:**
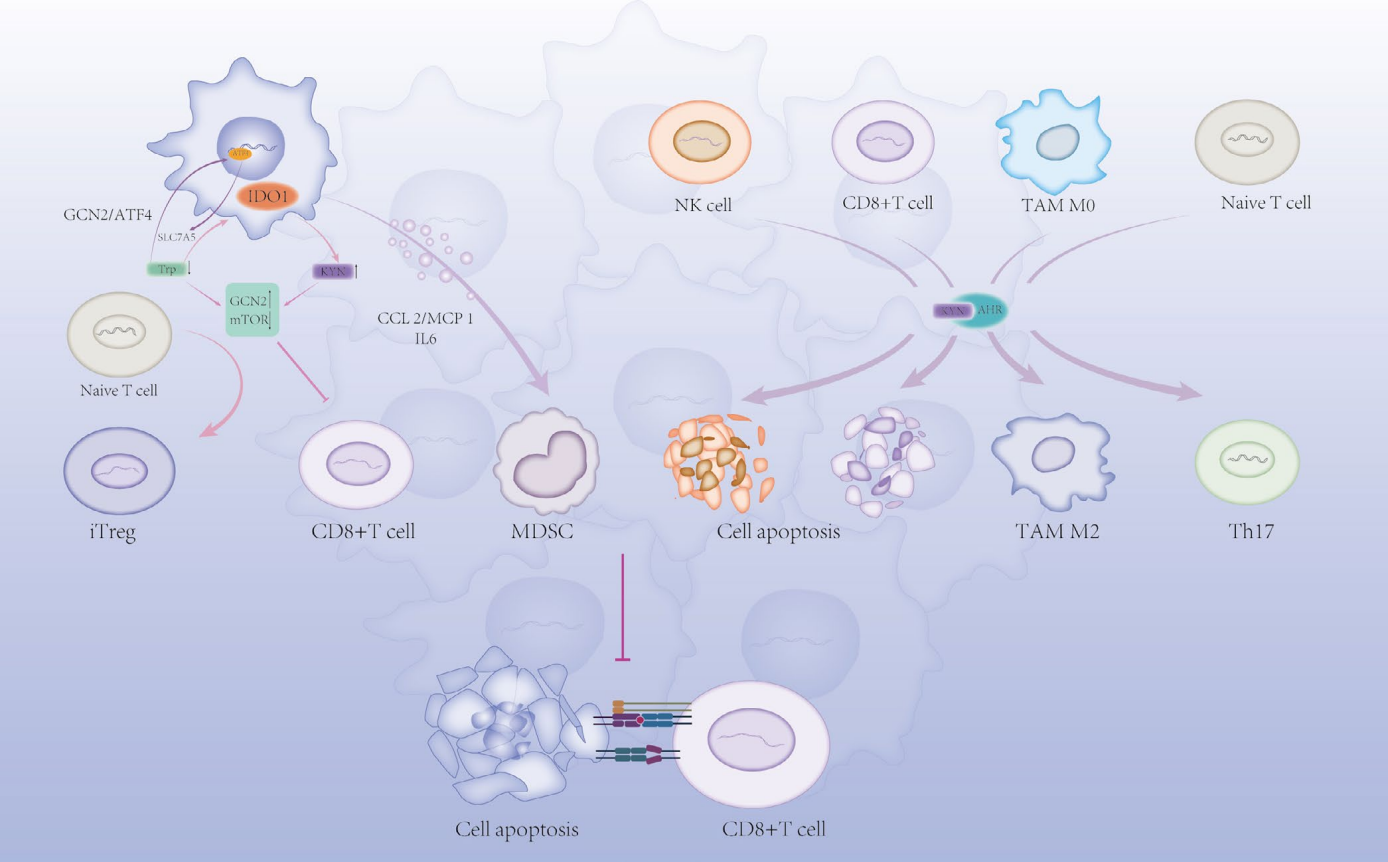
Immune suppressive effects of the kynurenine pathway. CCL2/MCP‐1, C–C motif ligand 2/monocyte chemoattractant protein 1; CD8+ T cell, cluster of differentiation 8‐positive T cell; GCN2, general control non‐derepressible 2; IDO1, indoleamine‐2,3‐dioxygenase 1; IL6, interleukin 6; MDSC, myeloid‐derived suppressor cell; mTOR, mechanistic target of rapamycin; NK cell, natural killer cell; SLC7A5, solute carrier family 7 member 5; TAM, tumor‐associated macrophage; Th17, T helper 17 cell; Treg, regulatory T cell.

Numerous studies [27] indicate that excessive activation of the Kynurenine Pathway (KP) is widely observed in lung cancer, gastrointestinal cancer, prostate cancer, glioma, melanoma, and pancreatic cancer, with a marked upregulation of IDO1, correlating with poor tumor prognosis. As key enzymes of the Kynurenine Pathway, IDO1 and TDO regulate immune responses by catalyzing the metabolism of tryptophan in various diseases. In tumor tissues, the heightened activity of IDO and TDO contributes to creating an immunosuppressive microenvironment, aiding tumor cells in evading immune surveillance. In tumor‐draining lymph nodes (TDLN), antigen‐presenting cells (APCs, such as dendritic cells) inherently expressing IDO1 induce iTregs, suppress T effector cells (Teffs), and result in systemic tumor immune suppression [28]. IDO1 overexpression in cancers is linked to the inactivation of cancer genes such as Bin1 [29] and p53 [30], BRAF mutations, or abnormal activation of the Mitogen‐Activated Protein Kinase (MAPK) pathway, which triggers Cyclooxygenase‐2 (COX‐2) upregulation, leading to Prostaglandin E2 (PGE2) autocrine release and eventually inducing IDO1 activation via Phosphoinositide 3‐kinase (PI3K) or Protein Kinase C (PKC) signaling pathways [31]. Moreover, in immune‐infiltrated tumors, antitumor factors like Interferon gamma (IFN‐γ), Tumor Necrosis Factor alpha (TNF‐α), Interleukin‐1 (IL‐1), Interleukin‐1 beta (IL‐1β), Interleukin‐6 (IL‐6), Programmed cell death protein 1 (PD‐1), Cytotoxic T‐lymphocyte‐associated protein 4 (CTLA‐4), and Transforming Growth Factor‐beta (TGF‐β) promote IDO1 expression [31, 32, 33]. IDO1 expression in tumors influences the expression of immune checkpoints such as Programmed death‐ligand 1 (PD‐L1) on Antigen‐Presenting Cells (APCs), leading to the suppression of T‐cell activity [34]. IDO1 establishes an immunosuppressive environment mainly through two mechanisms: 1. Tryptophan depletion 2. Immunosuppressive effects by metabolites (Table 1 and Figure 3).

**TABLE 1:** Efficacy of IDO1 in tumor biology.

Cellular process	Mechanism of action	Microenvironment or systemic	References
Immunosuppression	Tryptophan depletion promotes iTreg differentiation via GLK/mTOR and suppresses Teff	Microenvironment	[35, 36, 37]
	Tryptophan depletion activates GCN/ATF4, inducing SLC5A7 and further enhancing Teff tryptophan depletion	Microenvironment	[38, 39]
	Tryptophan depletion suppresses Teff ICOS/B7h, impacting immune signal amplification	Microenvironment	[40]
	The depletion of tryptophan promotes the expression of AHR and increases sensitivity to kyn in a GCN2‐independent manner, which may be related to Trp's antagonistic effect on AhR	Microenvironment	[41, 42, 43]
	Kyn/AHR shifts TAM from M1 to M2, enhancing tumor immunosuppression	Microenvironment	[44]
	Kyn/AHR induces an immunosuppressive APC phenotype, inhibiting NK and CD8+ cell activation and inducing apoptosis	Systemic	[44]
	Kyn/AHR promotes TGF‐β, IL‐6, and IL‐1 β expression, enhancing Treg differentiation and MDSC recruitment	Systemic	[45, 46, 47, 48]
	Kyn/AHR promotes IDO1 expression	Microenvironment	[49, 50]
	Kyn/AHR activates the MAPK pathway, promoting COL12A1 expression, contributing to ECM synthesis, and impacting lymphocyte immune infiltration	Microenvironment	[51]
	Non‐enzymatic activity (ITIM1): The TGF‐β‐IDO‐SHP‐1 axis upregulates TGF‐β and type I interferon expression, ultimately increasing IDO1 expression and maintaining a long‐term immunosuppressive phenotype in pDCs	Microenvironment and systemic	[52, 53, 54]
	Non‐enzymatic activity (ITIM2): In an inflammatory environment such as IL‐6, SOCS3 binds to SH2, inducing ubiquitin‐mediated IDO1 degradation	Microenvironment and systemic	[55, 56]
NAD+ synthesis	Oncogene URI downregulates KP, reducing NAD+ synthesis and decreasing DNA damage resistance, increasing cancer risk	Microenvironment	[57]
	Tumor c‐MYC mutations impair normal cell NAD+ synthesis, upregulating the salvage pathway of NAD+ synthesis	Microenvironment	[58]
Cell cycle regulation	IDO1 promotes tumor cell growth by inhibiting β‐catenin levels, reducing transcription of its target genes (e.g., cyclin D1 and Axin2) and cell proliferation	Microenvironment	[59, 60, 61]
	Elevated levels of IDO1 and its metabolite Kyn enhance CDC20‐mediated cyclin B1 degradation and CDK inhibition, independent of AhR activation	Microenvironment	[62]
	IDO1 upregulates MDM2 expression (the main negative regulator of p53), inhibiting p53 expression, thereby downregulating p21 and promoting cell growth; IDO1 upregulation is associated with decreased expression of pro‐apoptotic proteins PUMA and BAX, and increased expression of anti‐apoptotic proteins BCL2 and BCL‐XL	Microenvironment	[63]
	IFN‐γ‐IDO1‐kyn/AHR promotes transcription and expression of p27, inducing TRC dormancy, thus evading immune attack, while suppressing the JAK‐STAT1 apoptosis pathway	Microenvironment	[64, 65]
Tumor metastasis	IFN‐γ‐IDO1‐GCN/ISR counteracts IFN‐γ's anti‐angiogenic effects by upregulating IL‐6, promoting tumor angiogenesis	Microenvironment	[66, 67, 68]
	IDO1/Kyn‐AhR signaling pathway promotes tumor cell EMT	Microenvironment	[69]
	IDO1‐IL‐6/STAT3/PD‐L1 pathway promotes tumor cell EMT	Microenvironment	[9]
	IDO1‐JAK2/STAT3 pathway upregulates MMP‐2 and MMP‐9	Microenvironment	[70]
Ferroptosis	Kyn competes with cysteine at SLC7A11, causing pseudo‐starvation from cysteine deprivation, thereby enhancing GCN/ISR and mTOR‐mediated suppression of protein synthesis, diverting free cysteine to glutathione synthesis, which reduces lipid peroxidation	Microenvironment	[71]

#### 2.1.1 Effect of Tryptophan Depletion and Its Metabolites on Tumor Immunity

The first pathway (effect of tryptophan depletion): Increased IDO expression facilitates the conversion of Trp to Kyn, depleting Trp in the tumor microenvironment, resulting in several effects: (1) Unbound tRNA accumulation activates the general control non‐derepressible 2 (GCN2) kinase pathway, phosphorylating translation initiation factor eIF2α and inhibiting cap‐dependent translation [72]. At the same time, eIF2α activates transcription factor 4 (ATF4), which promotes the expression of tryptophan transporter SLC7A5 [38, 39]. Since tumor cells and effector T cells exhibit different abilities to upregulate SLC1A5 in a tryptophan‐depleted environment, tumor cells gain a competitive advantage in capturing Trp, helping to deplete Trp in the tumor microenvironment (TME) and suppressing effector T‐cell activation, thus inhibiting their immune function [58]. (2) As a metabolic regulation pathway, mTOR can decide whether to engage in protein synthesis or autophagy depending on amino acid levels. In a Trp‐deficient environment, mTOR activity is inhibited through the Glucokinase (GLK) pathway [35], leading to the conversion of CD4+Foxp3+Treg cells (iTreg) and suppression of effector T cells [36, 37]. Experiments suggest mTOR mediates ICOS expression on T cells, which interacts with B7h to promote immune infiltration of tumors. Reduced ICOS expression results in incomplete T‐cell activation and localized immune suppression [40]. Similarly, PKC activity is affected, with low Trp concentrations inhibiting its function, thereby weakening its ability to activate effector T cells [35]. (3) Tryptophan depletion also increases AhR expression, a mechanism that is independent of GCN2, and further enhances the activation of AHR by kynurenine [41]. Recent molecular docking experiments by Badawy revealed a strong binding between Trp and AhR. Combined with previous reports of Trp's downregulation and inactivation of AhR [42], as well as Trp's antagonistic effect on AhR activation, these findings [41, 43] suggest that Trp is likely an antagonist of AhR, and a reduction in Trp could lead to overactivation of AhR.

The second pathway (impact of tryptophan metabolites):

1. The tryptophan metabolite kynurenine (Kyn) induces tumor immune tolerance via the AhR. In tumor‐associated macrophages (TAMs), AhR regulates the shift between pro‐inflammatory M1 and anti‐inflammatory M2 macrophages [34]. Kynurenine produced by Antigen‐Presenting Cells (APCs) through AhR can block the activation of natural killer (NK) cells and CD8+ effector T cells, inducing apoptosis, while also suppressing T helper 17 (Th17) cells [28]. Myeloid‐derived suppressor cells (MDSCs) play a critical role in tumor immune suppression. AhR induces the recruitment and maintenance of MDSCs by promoting the expression of anti‐inflammatory cytokines such as TGF‐β, IL‐6, and IL‐1β, facilitating the differentiation of CD4+ cells into Treg cells, and enhancing MDSC suppression [45, 46, 47]. Moreover, AhR activation further promotes IDO1 expression, creating a positive feedback loop that intensifies the immunosuppressive effects [49, 50].
2. Other kynurenine metabolites, including 3‐hydroxykynurenine (3‐HK), 3‐hydroxyanthranilic acid (3‐HAA), and quinolinic acid, have been found to induce T‐cell apoptosis [73, 74].
3. The extracellular matrix (ECM) of tumors can form a barrier to prevent T‐cell‐mediated cytotoxicity [75, 76]. Xiang's experiments demonstrated that IDO1 upregulation activates the MAPK pathway via kyn/AhR, leading to increased expression of COL12A1. COL12A1 encodes the α1 chain of type XII collagen, which is involved in collagen cross‐linking and ECM synthesis, further contributing to immunosuppressive effects [51].

Notably, Badawy's molecular docking experiments revealed that Kyn did not bind to AhR, but rather activated AhR through its metabolite KYNA [42]. This result is supported by two factors: first, most previous experiments showing Kyn activation of AhR were conducted under conditions where Kyn concentrations far exceeded physiological levels (this will be discussed in detail below); second, prior studies [45] indirectly demonstrated Kyn's activation of AhR through the induction of AhR target genes (such as cytochrome P450‐dependent drug metabolism), without controlling for Kyn's potential metabolic transformation, where its downstream products like KYNA, AA, and 3‐HAA may also be involved. Although Kyn is not a suitable AhR ligand from a chemical structure perspective, Seok et al. found that Kyn in DMSO could spontaneously convert into a higher‐affinity aromatic condensation product [77]. Solvay's experiments also detected the same condensation derivatives in older Kyn solutions [41]. Therefore, it is possible that Kyn may activate AhR in a different form. However, this hypothesis requires further verification through cell and animal experiments.

The discovery of Trp as an AhR antagonist highlights its key role in maintaining bodily homeostasis and redefines the significance of Trp depletion—suggesting that this depletion may lead to a loss of protection against excessive AhR activation. This new concept not only overturns traditional understanding but also opens new pathways for future biological and clinical research. Future research should further explore the roles of Trp and Kyn in different cellular systems, particularly in the context of key enzyme expression or deficiency, to provide a solid scientific foundation for the development of novel therapeutic strategies.

#### 2.1.2 Non‐Enzymatic Functions of IDO1

In terms of tumor immune suppression, past studies have focused greatly on the enzymatic function of IDO1, but its non‐enzymatic function should not be overlooked. Mouse IDO harbors two Immunoreceptor Tyrosine‐based Inhibition Motifs (ITIMs), known as ITIM1 and ITIM2, which are highly conserved across mammals and positioned distantly from the catalytic domain [78].

**FIGURE 4:**
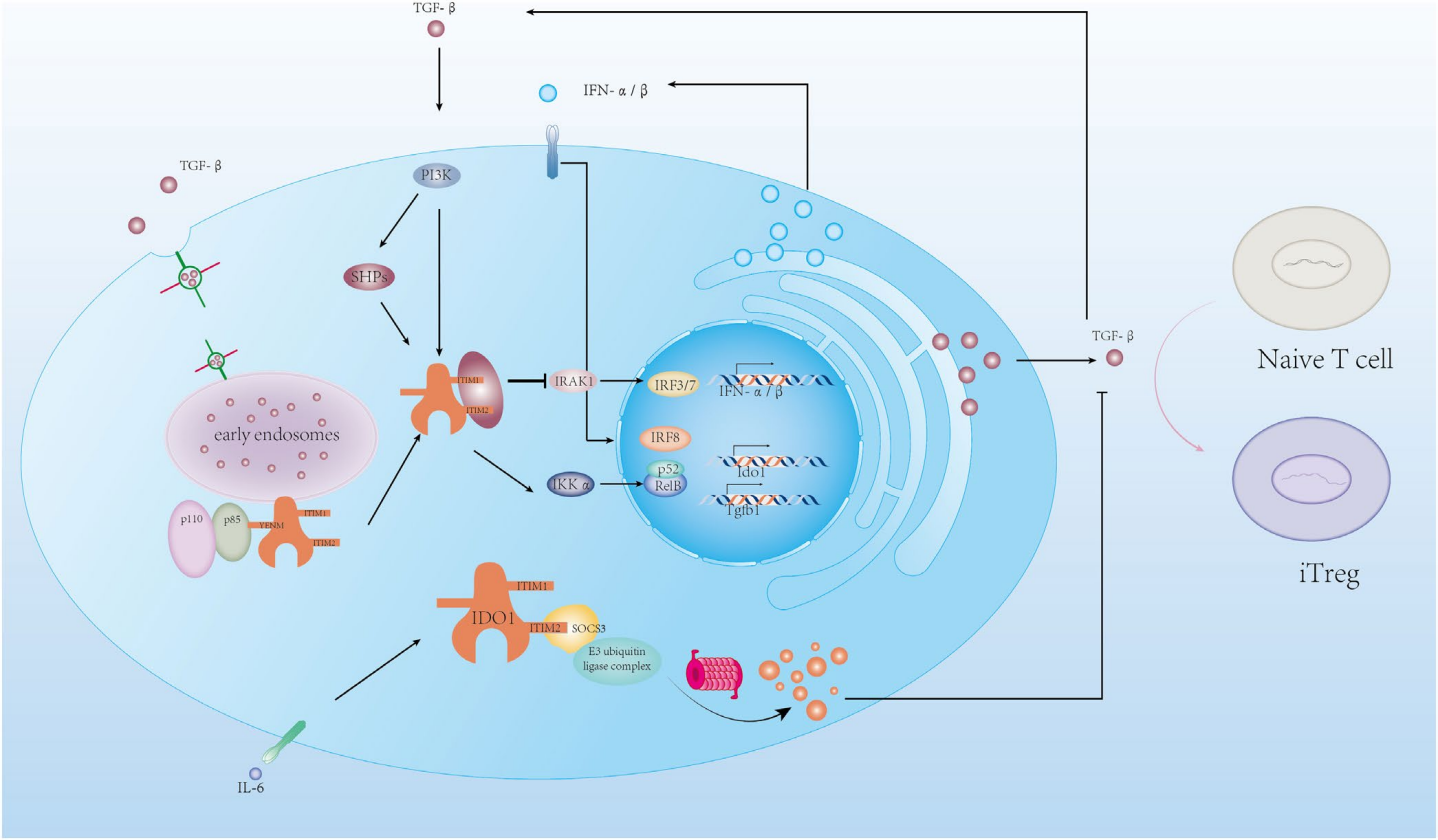
Non‐enzymatic functions of IDO1. The positive feedback of the TGF‐β‐IDO axis enhances IDO1 expression, resulting in persistent immunosuppression: In immunosuppressive settings, TGF‐β phosphorylates IDO1 via the PI3K pathway while inducing SHP expression. Once phosphorylated IDO1 binds with SHPs, it suppresses IRAK1 through protein–protein interactions, leading to the phosphorylation of kinase IKKα and activation of noncanonical NF‐κB pathways. Subsequently, this promotes the nuclear translocation of NF‐κB subunits p52 and RelB (markers of noncanonical NF‐κB signaling), which enhance the transcription of IDO1 and Tgfb1, culminating in increased expression of the corresponding proteins. The inhibition of IRAK1 removes its suppression on type I interferon release, promoting the expression of IFN‐α and IFN‐β via IRF3 and IRF7. IFN‐α/β promotes IDO1 expression by interacting with interferon regulatory factor IRF8 and the interferon‐stimulated response elements in the Ido1 promoter. In an inflammatory milieu, including cytokines like IL‐6, the elevated SH2 binding domain of SOCS3 interacts with the phosphorylated site of IDO1's ITIM2, recruiting the E3 ubiquitin ligase complex, leading to IDO1 degradation through ubiquitination and thereby triggering immune activation. In an immunosuppressive environment, the ITIMs (ITIM1 and ITIM2) and YENM domain of IDO1 are phosphorylated, with YENM activating PI3K by binding to its regulatory subunit P85, followed by the activation of the catalytic subunit p110, which anchors IDO1 to EEs, enhancing its non‐enzymatic function. AKT, protein kinase B; ERK1/2, extracellular signal‐regulated kinase 1/2; IFN‐α/β, interferon‐alpha/beta; IL‐6, interleukin 6; mTOR, mechanistic target of rapamycin; PI3K, phosphoinositide 3‐kinase; TGF‐β, transforming growth factor‐beta; Treg, regulatory T cell.

Plott's previous experiments demonstrated that under the intervention of the immunosuppressive cytokine transforming growth factor β (TGF‐β), plasmacytoid DCs (pDCs) can phosphorylate IDO's ITIM1 via the Frk kinase [55], then recruit SHP‐1 and SHP‐2 (upregulated by TGF‐β) to induce downstream pathways. SHP‐1 inhibits the kinase IRAK1, thus promoting noncanonical NF‐κB pathways, enabling pDCs to induce a higher frequency of Foxp3+ cells in CD4+ populations, leading to an immunosuppressive effect [52, 53]. Furthermore, the TGF‐β‐IDO‐SHP axis boosts the expression of TGF‐β and type I interferons, the latter of which binds to IDO1 promoters via IFN‐stimulated elements (ISREs) to regulate gene transcription, ultimately increasing IDO1 expression [54]. This upregulation creates a positive feedback loop to sustain the long‐term immunosuppressive phenotype of pDCs. These effects cannot be blocked by the inhibitor 1‐methyltryptophan (1‐MT) (Figure 4).

Curiously, ITIM2 has an opposite function [55]. When cytokines like IL‐6 are present, the SH2 binding domain of upregulated Suppressor of Cytokine Signaling 3 (SOCS3) binds to the phosphorylated site of IDO1's ITIM2, recruiting the E3 ubiquitin ligase complex, leading to IDO1 degradation via ubiquitination, and thereby activating immunity [56]. Although the phosphorylation site is far from the catalytic domain, Albini's experiment simulated the tyrosine phosphorylation state by replacing tyrosine residues with glutamic acid, revealing that IDO1 lost its enzymatic activity [55]. This indicates that IDO1's enzymatic and non‐enzymatic activities may not co‐occur, and phosphorylated IDO1 likely undergoes a conformational change at the catalytic site.

Recent studies have found that, in addition to ITIMs, IDO1 contains a binding site for the regulatory subunit p85 of PI3K, known as YENM, which also contains a tyrosine residue [79]. When this residue is phosphorylated, it binds to p85, recruiting the catalytic subunit p110 to regulate immune and anti‐inflammatory responses. Additionally, PI3K affects the transport of IDO1 proteins [80], anchoring them to early endosomes (EEs), which are rich in various effector proteins and receptors that facilitate signal transduction, including TGF‐β [81]. This promotes the TGF‐β‐IDO‐SHP signaling pathway. Epacadostat, a potent IDO1 catalytic inhibitor, demonstrated good efficacy in preclinical and early clinical trials [82] but did not show extra benefit when combined with pembrolizumab in Phase III randomized trials for metastatic melanoma [83]. One explanation proposed is that Epacadostat strengthens the interaction between IDO1 and SHP family tyrosine phosphatases while also facilitating IDO1's intracellular trafficking. Through binding with PI3K, IDO1 is anchored to EEs [84], which supports its immunosuppressive function—simultaneously inhibiting its catalytic activity and enhancing immune tolerance signaling.

The conflicting roles of ITIM1 and ITIM2 in IDO1 make sense in the context of responding to inflammatory signals, as pDCs, positioned at the forefront of immune responses, must quickly react to fluctuating pro‐inflammatory and anti‐inflammatory cues. In the tumor microenvironment, however, our understanding of how phosphorylated IDO1 influences immune signaling pathways is limited, and the response of other IDO1‐expressing cells, apart from pDCs, to their microenvironment remains to be studied. Previous research [33] has indicated that IL‐6 upregulates and sustains IDO1 expression in tumor cells, implying that IDO1 may act differently in response to identical signaling stimuli across different cell types. Considering both the catalytic and non‐catalytic roles of IDO1, the development of IDO1 inhibitors as anticancer therapies should account for both the inhibition of IDO1's catalytic activity and its impact on IDO1 signaling to enhance therapeutic efficacy.

#### 2.1.3 The Role of IDO1 Isoenzymes and Other Enzymes Associated With the Kynurenine Pathway on Tumor Immunity

TDO2 is expressed in various cancers [85], and Perez‐Castro's analysis of tumor mRNA in TCGA revealed a downregulation of IDO1 along with an upregulation of TDO in lung cancer [58]. Both TDO2 and IDO1 play key roles in the metabolism of Trp, and therefore, it is hypothesized that they play a role in tumor immunity. After IDO1 silencing, TDO2 may function as a compensatory mechanism to sustain tumor growth. Experiments [45, 85] Reversal of tumoral immune resistance by inhibition of tryptophan 2,3‐dioxygenase in vivo and in vitro have demonstrated that TDO2 influences T‐lymphocyte function by inhibiting their proliferation. TDO2 promotes M2 macrophage activation by upregulating IL‐8, triggering the AKT/GSK3β pathway, thereby supporting the growth of esophageal squamous cell carcinoma [86]. TDO2 functions similarly to IDO1, as the metabolism of Trp can reach levels sufficient to activate AhR [87]. Moreover, TDO‐positive cells suppress IFN‐γ production through TDO2‐mediated antibacterial mechanisms [88], potentially linking this effect to immune suppression in the tumor environment.

IDO2, the isoenzyme of IDO1, is overexpressed in non‐small cell lung cancer, pancreatic cancer, and cervical cancer [89]. However, because of its low affinity for tryptophan [90], its IDO1‐like effects are less noticeable, and its role in immune suppression remains controversial. Research has shown that IDO2 induces the activation of autoreactive T and B cells, facilitating autoantibody production, indicating its pro‐inflammatory role. Further studies [91] have revealed that when IDO1 and IDO2 are co‐expressed, competition for heme binding occurs, thereby negatively regulating IDO1. Whether IDO2 functions similarly in a tumor context requires further investigation.

Downstream metabolic enzymes of Kyn are also crucial for the function of the KP, as Kyn's metabolic products play corresponding roles in tumor immunity. On the other hand, Kyn metabolism implies a reduction in its concentration, which will inevitably impact its associated functions. The three main downstream metabolic enzymes of Kyn mentioned above are KMO, KUNY, and KAT.

KMO is largely downregulated in most tumors, but it is upregulated in inflammatory environments under stimulation from factors like IFN‐γ [92, 93]. This indicates that in immune‐stimulated hot tumors, KMO upregulation causes increased 3‐HK expression. As previously mentioned, 3‐HK suppresses effector T cells. The upregulation of IDO1 in an inflammatory environment also compensates for the Kyn consumed by elevated KMO.

KAT shows different responses to the same stimuli across tumor types; for instance, IFN‐γ induces KAT expression in head and neck squamous cell carcinomas, but it decreases in glioblastoma multiforme [94]. Given KAT's higher Km, its impact on the Kyn:Trp ratio is less pronounced than that of the other two enzymes [95]. However, when Kyn accumulates to a certain level in the microenvironment, this enzyme's catalytic reaction can still proceed [94]. As a metabolic product of KAT, KYNA has multifaceted effects on tumors, promoting antitumor immunity while also inhibiting tumor cell cycle progression and invasiveness. In breast cancer [96, 97], KYNA binds to AhR to promote IL‐6 expression (enhanced binding of AhR with RelB leads to increased IL‐6 transcription). IL‐6 regulates immunity in a bidirectional manner. Fisher's review summarized IL‐6's antitumor immune effect, which is achieved by influencing lymph node activation and metastasis, as well as immune infiltration in the tumor microenvironment [98]. On the other hand, IL‐6 partially relies on autocrine IL‐10 mechanisms and inhibits NF‐κB activity and alters NF‐κB subunit composition, thereby synergistically inhibiting CCR7 expression in DCs, which affects their migration ability and prevents effective activation of Teff [99]. In breast tumor cells, co‐treatment with KA and IL‐1β significantly induces IL‐6 expression, and this induction depends on the expression of AhR [97]. In lung cancer [33], IL‐6 activates STAT3 to induce IDO1, which in turn produces Kyn and KYNA to activate AhR and induce IL‐6 expression. This establishes an AhR‐IL6‐STAT3 loop, which maintains the continuous activation of the KP pathway and suppresses antitumor immunity. KYNA, acting as an AhR ligand, has been shown to have a higher affinity and stability than Kyn [97]. Badawy recently suggested through molecular docking experiments that Kyn might activate AhR via KYNA [42]. Regardless of the situation, it is certain that KYNA can promote tumor immune evasion via AhR [100]. Additionally, KYNA may inhibit tumor cell proliferation upon receptor activation and influence tumor metastasis. This will be further discussed below.

KYUN is elevated in certain autoimmune diseases in humans, and in the process of Kyn degradation, it reduces Foxp3 expression, thereby enhancing local immune responses [101]. Unlike the immune suppression observed in tumor microenvironments, IDO1 upregulation in the pro‐inflammatory environment of these inflammatory diseases does not significantly reduce inflammation, possibly due to KYUN expression. Harden's analysis of cancers found that IDO1 and TDO2 are preferentially upregulated over KYUN, whereas the opposite occurs in pro‐inflammatory environments. Thus, compared to inflammatory diseases, cancers show a significantly higher IDO1‐TDO2 ratio. Upregulating KYNU and degrading kynurenine to weaken kyn‐AhR signaling may be a reliable way to control tumor immunosuppression. For instance, overexpressing KYNU in chimeric antigen receptor (CAR)‐T cells reduces immunosuppressive Kyn in the tumor microenvironment, enhancing CAR‐T cell efficacy [102]. In mouse models of melanoma, breast cancer, and colon cancer, PEGKYNase (modified with polyethylene glycol (PEG) to enhance enzyme stability and half‐life) can reduce serum Kyn, increase AA levels, and simultaneously increase the number of CD8+ T cells infiltrating the tumor microenvironment, reducing tumor volume [103].

Interestingly, León‐Letelier and colleagues discovered that higher levels of KYNU expression in lung adenocarcinoma (LUAD) and pancreatic cancer tissues are linked to an immunosuppressive tumor microenvironment [104, 105]. Upregulation of KYNU can increase the number of Tregs and the expression of immune checkpoint blockade genes PD‐1 and PD‐L1. These studies suggest that the immunosuppressive role of KYNU is tumor‐specific [104].

Heng's research may provide an explanation for this contradiction [106]. KYNU breaks down Kyn and 3‐HK into AA and 3‐HAA. Studies [107, 108, 109, 110, 111] have shown that 3‐HAA can suppress nitric oxide (NO) production in macrophages, thereby reducing their tumor‐killing function, inducing apoptosis in cytotoxic T cells and Th1 cells, weakening immune‐mediated tumor destruction, and enhancing tumor immunosuppression by modulating HLA‐G expression on dendritic cells and promoting Treg cell differentiation. AA, however, has not been observed to impact immune cell activity or function in existing research. Heng found that KYNU's substrate affinity changes under inflammatory stimulation; for example, 3‐HAA synthesis is significantly increased in breast cancer cells under IFN‐γ stimulation. The production of 3‐HAA also depends on environmental pH and iron ion availability, factors that may limit the conversion from AA to 3‐HAA [106]. Thus, variations in the tumor microenvironment result in different substrate preferences for KYNU, thereby affecting tumor immunosuppression.

Badawy's review concluded that most tumors prefer to upregulate enzymes upstream of Kyn and downregulate downstream enzymes, leading to higher Kyn levels and enhanced immunosuppressive effects [7]. Analysis of key enzymes in the Kyn metabolic pathway revealed their roles in immunosuppression, with KYNU appearing to serve as an “on–off” switch for immune suppression. Tumors undoubtedly favor Kyn, but different tumor types, owing to unique disease patterns, affect tryptophan metabolic enzymes in varied ways. These differences could result in opposite roles for the same enzyme in distinct tumors, making it necessary to examine enzyme alterations and metabolite levels in different tumors.

#### 2.1.4 Tryptophan Acquisition by Tumor Cells

Tumor cells' requirement for essential amino acids causes upregulation of essential amino acid transporters, a characteristic shared by many cancers. Large neutral amino acid transporters (LAT), as Trp transporters, are also upregulated in most tumors. Studies have identified SLC7A5 (LAT1), SLC1A5, SLC7A11, and SLC6A14 as Trp transporters [7, 112]. Perez‐Castro et al. [58] found that SLC7A5 and SLC1A5 are upregulated in 13 types of tumors, while SLC6A14, distinct from the other two transporters, is ideal for tumor growth and development due to its strong concentration capacity and broad‐spectrum amino acid transport ability [113]. Its expression is upregulated in multiple epithelial‐derived cancers [114, 115]. Additionally, another Trp transporter, distinct from the L‐type system and expressed by IDO‐positive tumor cells, significantly increases Trp uptake into the tumor under low Trp concentrations [116]. Thus, tumor cells hold an advantage in Trp acquisition over other cells in the same microenvironment due to these transporters.

Research has also determined that Kyn is transported through the system L transporter in activated T cells, with SLC7A5 recognized as a crucial transporter [117, 118]. SLC7A5 not only transports Kyn but also large neutral amino acids like leucine. Therefore, Kyn competes with essential amino acids for SLC7A5, influencing leucine availability, which in turn affects mTORC1 pathway activation and c‐Myc expression. Myc, a critical metabolic reprogramming factor [119], coordinates metabolic gene transcription during T‐cell activation to support growth and proliferation, also inducing SLC7A5 and SLC1A5 in multiple cell lines [120, 121]. Consequently, this competition ultimately inhibits T‐cell activation, proliferation, and metabolism, producing an immunosuppressive effect.

As previously mentioned, Trp depletion and its decomposition products have synergistic effects. For instance, tumors expressing IDO1 have an advantage in acquiring Trp. After IDO1 consumes Trp, the subsequent reduction of Trp and Kyn production in the microenvironment enhances Trp transporter activity, further depleting Trp in immune cells and inducing apoptosis. Despite the upregulation of SLC7A5 in effector T cells under inflammatory conditions stimulated by the inflammatory factor IFN‐γ, the large amount of Kyn produced by tumors competes for SLC7A5 binding (partially explaining the reduced Trp acquisition in effector T cells within the tumor microenvironment), affecting effector T‐cell growth and proliferation [118]. Trp depletion increases the sensitivity of AhR to Trp metabolites like Kyn, potentially linked to upregulation of AhR and the LAT1 transporter for Trp and Kyn under Trp deprivation [41]. However, other studies suggest that under IDO1 overexpression, Trp levels do not reach the GCN2 activation threshold, with the primary effect arising from IDO1 metabolites [122]. Sinclair et al. observed that plasma kynurenine (Kyn) concentrations seldom surpass 3 μM, while concentrations of other system L substrates in plasma (such as leucine, tryptophan, and methionine) range from 20 to 100 μM, with Km values roughly one‐tenth of Kyn's [118, 123]. This suggests that Kyn would require concentrations significantly above physiological levels to effectively compete with other high‐affinity substrates. Additionally, in vitro experiments demonstrating AhR activation used Kyn concentrations (50–100 μM) exceeding normal physiological plasma Kyn levels [45, 124, 125], suggesting it may be difficult for Kyn to act in vivo; however, the differences between tumor microenvironments and normal plasma composition may make it possible. For instance, Ho et al.'s study showed that glucose concentrations measured in tumors were 10 times lower than in matched spleen or blood samples [126]. Earlier, we mentioned efficient Trp transporters that promote Trp accumulation in tumor cells, establishing the foundation for Trp depletion in tumors. Opitz et al. measured a Kyn concentration of 37 μM in a U87 xenograft tumor model [45]. If essential amino acids drop tenfold similar to glucose, IDO1 in the tumor environment could elevate Kyn tenfold, reaching the competitive level for SLC7A5 and effectively activating AhR. In the previous text, we mentioned that Badawy's molecular docking experiment found that Kyn did not bind to AhR, and related studies suggest that Kyn activates AhR indirectly through KA [42]. KAT‐mediated metabolism of Kyn is more dependent on Kyn concentration. Previous research observed that when healthy individuals ingested small amounts of tryptophan (1.15 g, approximately 16 mg/kg), their plasma Kyn levels rose by 47% [127]. This change led to a significant 209% increase in KA concentration. This indicates that the change in KA concentration is amplified based on the change in Kyn concentration. In the tumor microenvironment, an increase in kyn concentration may lead to a more substantial rise in KYNA, which has a higher affinity and stability than Kyn, and its resulting AhR activation effect should not be overlooked. Thus, more detailed quantitative metabolite analysis in the tumor microenvironment may clarify these mechanisms in future research (Figure 4).

### 2.2 Kynurenine Pathway's Impact on Tumor Growth and Cell Cycle

Research [61] has shown that tumors expressing IDO1 grow more rapidly in immunodeficient SCID/beige mice, indicating a non‐immune mechanism in controlling cell growth. Tumors with IDO1 gene silencing are more sensitive to apoptosis, and the proportion of their cells in the S and M phases is significantly reduced compared to cells expressing IDO. The downregulation of IDO1 correlates with the decreased expression of multiple genes regulating G1/S transition, S phase, G2/M transition, checkpoints, and M phase. In human colorectal cancer cells, IDO1 inhibition reduces nuclear activated β‐catenin levels, diminishes transcription of its target genes (e.g., cyclin D1 and Axin2), and hampers cell proliferation. Exogenous kynurenine and quinolinic acid (QA) administration activate β‐catenin, promote human colorectal cancer cell proliferation, and accelerate tumor growth in mice [59, 60]. Liu's experiments confirmed that in non‐immune environments, elevated levels of IDO1 and its metabolite Kyn in colon cancer cells significantly promote CDC20 transcription, and inhibiting IDO induces mitotic death through CDC20 [62]. Kyn enhances CDC20‐mediated cyclin B1 degradation and CDK suppression independently of AhR activation, accelerating cell cycle progression and proliferation. Interestingly, Kyn was observed to translocate to the nucleus, which may be associated with the activation of other transcription factors. In diffuse large B‐cell lymphoma (DLBCL), inhibiting IDO1 restores p53 expression by downregulating MDM2, the principal negative regulator of p53. p53 triggers cell cycle arrest and apoptosis through p21 transcription, and inhibiting IDO1 raises pro‐apoptotic proteins PUMA and BAX expression while decreasing anti‐apoptotic proteins BCL2 and BCL‐XL [63]. Silencing IDO2 with siRNA inhibits melanoma cell growth, induces G1 phase cell cycle arrest, and promotes apoptosis. In vivo, IDO2 silencing via shRNA delays tumorigenesis and suppresses tumor growth, indicating a role for IDO2 in promoting the tumor cell cycle [128]. Interestingly, although TDO promotes tumorigenesis in gliomas, breast cancer, melanoma, cervical cancer, and colorectal cancer, in hepatocellular carcinoma, TDO2 overexpression upregulates p21 and p27 protein levels and decreases CDK2 and CDK4 protein expression, leading to G1 phase cell cycle arrest [129].

**FIGURE 5:**
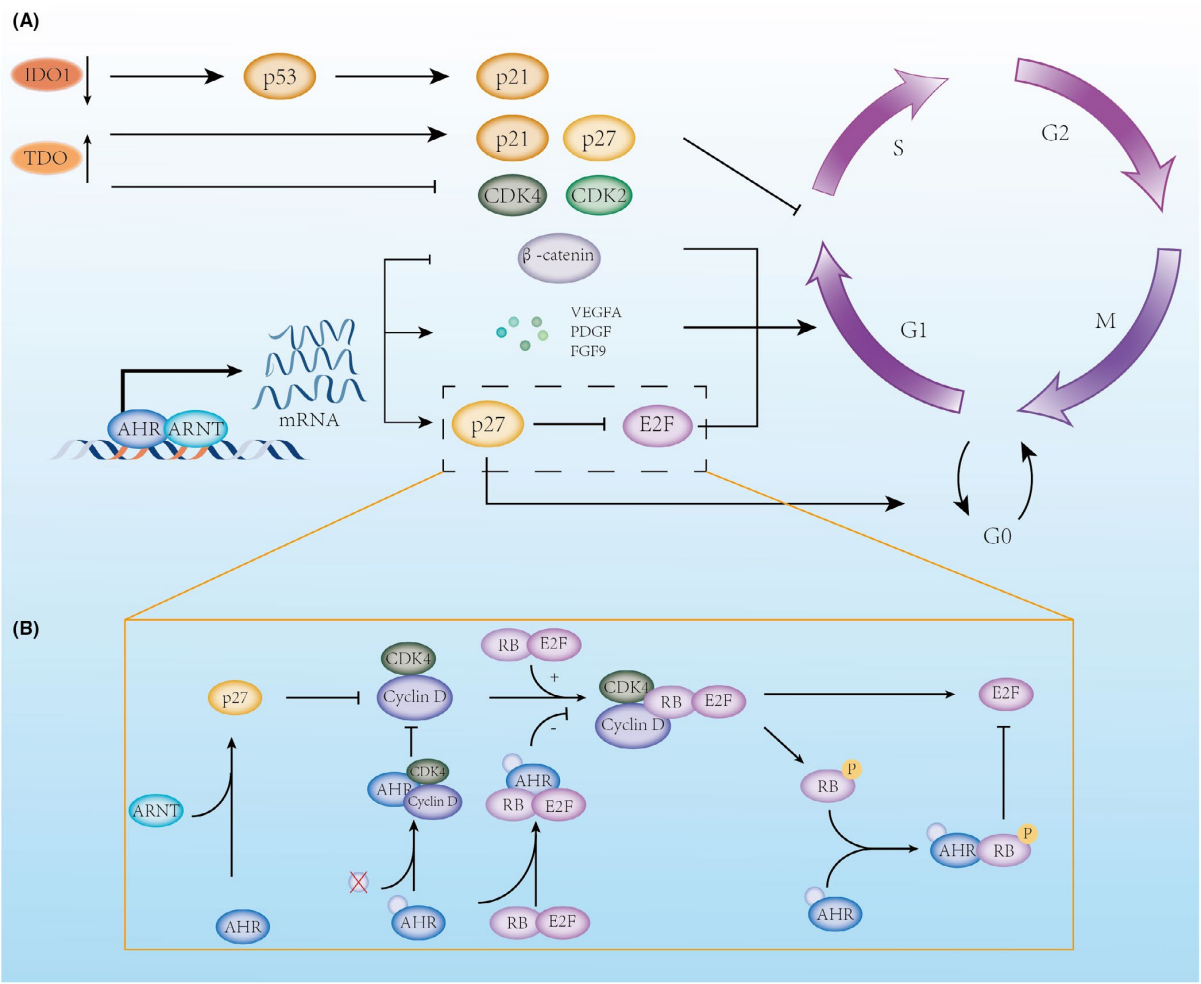
Kynurenine pathway's impact on tumor growth and cell cycle. (A) IDO1 silencing restores p53 expression by downregulating MDM2 (a key negative regulator of p53), and p53 induces cell cycle arrest and apoptosis by transcribing p21. p53 causes cell cycle arrest and apoptosis through p21 transcription. TDO2 overexpression increases the expression of p21 and p27 proteins while reducing CDK2 and CDK4, causing cell cycle arrest at the G1 phase. (B) Different AhR ligands produce different effects when binding to AhR. (1) AHR induces mitogenic growth factor gene expression, facilitating tumor cell proliferation. (2) After binding with ligands, AhR degrades β‐catenin via ubiquitination, suppressing cyclin expression. (3) Upon receiving division signals, CDK4/6 binds with cyclin D1, leading to RB phosphorylation (inactivation), reducing RB‐E2F complex formation, which increases E2F transcriptional activity, promoting cell cycle gene expression and progression. AhR increases p27 expression, preventing RP phosphorylation and E2F release, causing G1 phase arrest. Unbound and ligand‐bound AhR interact with CDK4‐Cyclin D and RB‐E2F to inhibit E2F release, respectively. Ligand‐free AhR impacts E2F activity by binding to pRB. In tumor stem cells, p27 binds to p‐STAT1, inhibiting the IFN‐γ‐induced JAK–STAT apoptosis pathway, promoting entry into the G0 phase and evasion of early tumor immunity. AHR, aryl hydrocarbon receptor; ARNT, aryl hydrocarbon receptor nuclear translocator; CDK2, cyclin‐dependent kinase 2; CDK4, cyclin‐dependent kinase 4; E2F, E2F transcription factor; FGF9, fibroblast growth factor 9; G0, G0 phase (resting phase of the cell cycle); G1, G1 phase (first gap phase); G2, G2 phase (second gap phase); IDO1, indoleamine‐2,3‐dioxygenase 1; IL‐6, interleukin 6; M, M phase (mitosis); mRNA, messenger RNA; P, phosphorylation; p21, cyclin‐dependent kinase inhibitor 1A; p27, cyclin‐dependent kinase inhibitor 1B; p53, tumor protein p53; PDGF, platelet‐derived growth factor; RB, retinoblastoma protein; S, S phase (synthesis phase); TDO, tryptophan 2,3‐dioxygenase; VEGFA, vascular endothelial growth factor A; β‐catenin, beta‐catenin.

Liu's study [64] revealed that in tumor‐repopulating cells (TRCs), IFN‐γ‐induced IDO1 promotes p27 transcription and expression via the kyn/AhR pathway. As a cell cycle inhibitory protein, p27 induces TRCs into dormancy, protecting them from immune attacks. In the process of tumor immunity, IFN‐γ induces apoptosis through the JAK‐STAT1 pathway. Experiments [65, 130] revealed that p27 binds to p‐STAT1, preventing its nuclear translocation, thereby avoiding apoptotic signaling via caspases 2, 3, and 7, and redirecting the IFN‐γ signal toward the kyn/AhR pathway. The inconsistent behavior between TRCs and regular tumors may be due to the differential expression patterns of IDO1 and AhR in tumor stem cells. Since cellular dormancy requires high signaling, the low expression of IDO1 and AhR in tumor cells fails to generate sufficient p27 and other dormancy‐related proteins, leading to a different response to IFN‐γ. The early activation of NK and effector T cells in tumors provides IFN‐γ, helping TRCs enter dormancy and avoid immune attacks. Previous research [131, 132, 133, 134] showed that the PGE2 pathway regulates the transition of tumor cells from dormancy to proliferation, and inhibiting this pathway could awaken dormant tumor cells and boost their sensitivity to immunotherapy. The autocrine COX‐2/PGE2 pathway persistently activates IDO1, which might partially explain its role in promoting dormancy in tumor cells (Figure 5).

KYNA exerts opposing effects on tumor cell growth and the cell cycle, with recent studies indicating that KYNA inhibits the proliferation of human glioblastoma T98G cells [135], colon cancer [136], and kidney cancer cells [137]. In colon cancer HT‐29 and kidney cancer Caki‐2 cells, KYNA inhibits key CDK complexes within the cell cycle by upregulating p21Waf1/Cip1 expression, leading to cell cycle arrest and antiproliferative outcomes [137, 138, 139]. In Caki‐2 kidney cancer cells, KYNA affects the inhibition of Rb phosphorylation and inhibits the signaling kinase p38 MAPK. The phosphorylation of the former protein ultimately affects the expression of G1‐to‐S‐phase cyclins, while the latter is also involved in G1/S and G2/M checkpoint cell cycle control through multiple pathways [140]. Previous studies [140] have found that KYNA, as an ionotropic glutamate receptor antagonist, reversed the stimulatory effect of glutamate on the proliferation of glioma T98G cells. In some studies [141, 142, 143], AhR has also shown the ability to inhibit tumor growth. Therefore, it remains to be further studied whether KYNA's inhibitory effect on human glioblastoma T98G cells, human colon cancer, and renal cancer cells occurs through the AHR pathway.

Previous studies have shown that AhR's effect on tumor cell growth varies depending on the tumor type, and its influence on the cell cycle has presented conflicting results. AhR can function as a transcription factor, and upon agonist activation, it enhances the expression of cell cycle inhibitors p21 and p27 [144, 145], which, after binding to cyclin D1 or E, suppress RB phosphorylation, resulting in cell cycle arrest at the G1 phase. Moreover, after binding with ligands, AhR interacts with unphosphorylated RP and β‐catenin [146, 147, 148]. RP binding inhibits its phosphorylation and prevents E2F release, leading to G1 phase arrest, while β‐catenin is degraded through AhR‐mediated ubiquitination, suppressing cell cycle‐dependent gene expression and producing similar results. When AhR is not bound to its transport proteins, it binds to pRB to create a co‐repressor that inhibits E2F‐mediated transcriptional activation by displacing histone acetyltransferase p300, leading to the suppression of S‐phase gene expression [149]. AhR facilitates cell proliferation primarily by inducing the expression of mitogenic growth factor genes [150], including vascular endothelial growth factor A (VEGFA), platelet‐derived growth factor (PDGF), epiregulin, amphiregulin, and fibroblast growth factor 9 (FGF9). These factors promote M‐phase entry and enhance cell proliferation. Furthermore, evidence suggests that unbound AhR binds to cyclin D and cyclin‐dependent kinases 4 (CDK4), preventing them from phosphorylating RP, but this interaction is lost after AhR ligand supplementation, allowing cyclin D and CDK4 to regain function and support cell proliferation.

Regarding AhR ligands from tryptophan metabolism, we found a similar contradiction: KYN and KYNA, as endogenous AhR ligands, suppressed cyclin D1 and CDK4 protein levels in A375 cells and decreased Rb protein phosphorylation in melanoma cell lines, yet KYNA showed an opposing effect on protein levels and cell cycle regulators in metastatic RPMI7951 cells [151]. Katarzyna's later experiments [152] also revealed that KYN and KYNA at a concentration of 5 mM increased cyclin D1 levels in SK‐MEL‐3 cells, even though both promoted p21 Waf1/Cip1 and p27 Kip1 protein expression, exhibiting antiproliferative effects on tumors. In addition, the tryptophan metabolite xanthurenic acid (a tryptophan metabolite) can activate AhR and promote the growth of LN‐308 glioma cells in CD1nu/nu mice [45]. Additionally, it is important to note that Badawy reported a series of indole metabolites binding to AhR through molecular docking experiments. The data do not always reflect the in vivo situation, as there is no significant correlation between the docking scores and EC50 values of the 12 Trp metabolites. The differences in potency between the metabolites may be due to metabolism and other factors, such as substrate concentration, enzyme reactions, redox status, competition from other ligands, and protein binding. Therefore, this may also lead to differences between in vivo and in vitro study results [42].

IDO1's impact on tumor growth and apoptosis has strengthened the case for using its inhibitors in cancer therapy. Differences in IDO1 expression between common tumor cells and cancer stem cells offer insights for optimizing cancer treatment strategies. In early‐stage tumors, reducing tumor‐regenerating cells as much as possible is key, while in late‐stage tumors, promoting their dormancy might extend survival. The role of endogenous tryptophan metabolites in activating AhR and their impact within tumors still requires further investigation. Because in vitro cultures do not replicate the complex in vivo tumor microenvironment, involving immune, stromal, and tumor‐associated signaling cells, the action of AhR ligands derived from tryptophan metabolism requires more accurate in vivo verification.

### 2.3 Kynurenine Pathway's Impact on NAD+ Synthesis

NAD+ is composed of adenine and nicotinamide. These two units are connected by a phosphodiester bond, forming NAD+.

NAD+ serves as a coenzyme in cellular energy metabolism, oxidative stress, and DNA repair [57], while also acting as a substrate for enzymes like the sirtuin protein family, Poly ADP‐ribose polymerases (PARPs), and Cyclic ADP‐ribose synthases (cADPRSs), associating it with aging‐related diseases, immune dysfunction, and cancer [153]. Studies show that NAD+ is synthesized in the human body primarily through three pathways: 1. the de novo pathway from QA; 2. the Preiss‐Handler pathway from nicotinic acid; and 3. the salvage pathway from nicotinamide. The first pathway, as previously mentioned, involves tryptophan metabolism into QA, followed by three enzymatic steps to form NAD+.

1. Studies [57] show that in liver cells, the oncogene URI downregulates the de novo NAD+ synthesis pathway via the kynurenine pathway, diminishing DNA damage repair capacity and raising the risk of tumor development.
2. After tumorigenesis, c‐MYC mutations promote the salvage pathway, suppress NAD+ production through the tryptophan and Preiss‐Handler pathways, disrupting NAD+ metabolism in normal cells while preserving essential NAD+ for tumor cells, thereby protecting them [58]. Additionally, increased NAD+ production in tumors can enhance the expression of PARPs and sirtuins [153, 154]. The former aids tumor cells in repairing DNA damage, thereby preventing various forms of programmed cell death, while the latter, specifically SIRT1 and SIRT2, reduce tumor suppressors and stabilize oncogenes. Therefore, the NAD+ generated through the tryptophan metabolic pathway plays a crucial role, both prior to tumorigenesis and in the presence of existing tumors.

### 2.4 The Impact of the Kynurenine Pathway on Tumor Invasiveness

Tumor invasiveness is related to many factors, such as epithelial–mesenchymal transition (EMT), extracellular matrix degradation, angiogenesis, and changes in the tumor microenvironment.

Previous studies have found that the abnormal expression of TDO2 and IDO1 is related to tumor invasiveness. Levina [61] discovered that breast tumors expressing IDO1 in mice tend to metastasize to the lungs. The upregulation of IDO1 expression is positively correlated with myometrial invasion, lymph node metastasis, and lymphovascular space involvement [155]. In triple‐negative breast cancer [156], abnormal TDO2 expression increases the tumor's resistance to the absence of a basement membrane, inhibiting related programmed apoptosis when tumor cells detach from the basement membrane.

#### 2.4.1 IDO1 Serves as a Crucial Interface Between IFN‐γ and IL6 in Tumor Angiogenesis, Steering the Inflammatory Milieu Toward Promoting Vascular Development

Saga's experiment [157] found that IDO1 promotes angiogenesis and enhances peritoneal dissemination of ovarian cancer. Smith's study [66] discovered that silencing IDO1 in a KRAS‐induced lung adenocarcinoma model reduced the number of blood vessels in both tumors and non‐tumor tissues. IL6 expression also significantly decreased with IDO1 silencing, but angiogenesis was restored after IL‐6 supplementation. Their team confirmed this finding in follow‐up studies [68]. In the OIR model, IL‐6 silencing reduced angiogenesis, demonstrating that IDO1 is a crucial regulatory node between IFN‐γ and IL6, promoting IL6 production through the GCN2‐mediated Integrated Stress Response (ISR) pathway [67]. This balances IFN‐γ's anti‐angiogenic effects, supporting angiogenesis under inflammatory conditions. The direct pro‐angiogenic effect of IL‐6 cannot be overlooked. IL6 promotes angiogenesis via VEGF‐independent mechanisms [158] and is associated with matrix metalloproteinases (MMPs), which facilitate endothelial cell sprouting by degrading the vascular extracellular matrix [159]. In patient‐derived nasopharyngeal carcinoma, IL‐6/NOS2 signaling regulates MMP‐9 and MMP‐2 dependent metastasis [160].

The specifics of IDO1‐induced angiogenesis still require further study. Blaschitz et al. have identified that endothelial cells express IDO1, which may affect blood vessels through downstream tryptophan metabolites or local tryptophan depletion [161]. The tryptophan depletion effect has been confirmed in Dey's experiment, but their study did not indicate whether tryptophan metabolites affect angiogenesis [67]. Previous studies have found that endothelial cells expressing IDO1 in inflammation relax blood vessels through kynurenine. Zhang's recent experiment demonstrated that in a neonatal mouse cardiac deficiency model, Kyn produced by cardiomyocytes was transported to endothelial cells, promoting aryl hydrocarbon receptor translocation, which enhanced the expression of angiogenic genes like VEGF‐A [162]. Previous studies have shown that AhR regulates angiogenesis through VEGF activation in endothelial cells and TGF‐β inactivation in the stroma [45]. This provides a reference for studying IDO1‐related angiogenesis in the tumor context, and exploring its underlying mechanisms could offer a basis for new drug development or more rational and effective combination therapies. IDO1's pro‐angiogenic activity in both tumor and non‐tumor tissues helps create a favorable microenvironment for tumor initiation, later promoting tumor growth and metastasis. Further research may examine whether differential IDO1 expression in normal tissues correlates with metastatic potential.

As an isoenzyme, IDO2 has no confirmed pro‐angiogenic role, whereas TDO has been observed in the neovasculature of liver cancer, glioblastoma, melanoma, and bladder cancer [163]. It may work synergistically with IDO1, but the extent of its pro‐angiogenic effect is yet to be clarified. Furthermore, studies [45, 164, 165] have shown that TDO expression increases under the influence of IL‐1β, which upregulates IL‐6 expression and promotes angiogenesis in endometrial stromal cells and glioma cells.

#### 2.4.2 Overexpression of TDO and IDO1 Affects the Tumor Extracellular Matrix (ECM) and Enhances Epithelial‐Mesenchymal Transition (EMT), Increasing Tumor Cell Motility, Promoting Distant Metastasis, and Resistance to Anoikis

The zinc finger transcription factor Slug is a target gene of AhR. Slug plays a role in epithelial–mesenchymal transition (EMT) by increasing the expression of vimentin and inhibiting the mRNA expression of E‐cadherin [166]. TCDD (2,3,7,8‐tetrachlorodibenzo‐*p*‐dioxin) is a widely recognized AhR agonist with high affinity for AhR [167]. TCDD affects Slug expression via AhR, thereby participating in the regulation of EMT in breast cancer cells. However, another AhR agonist, MEHP, competitively antagonizes TCDD, reducing its ability to promote tumor EMT [168]. Serum Response Factor (SRF), as a transcription factor, promotes epithelial cell migration and invasion in prostate cancer, gastric cancer, cervical cancer, and hepatocellular carcinoma and is associated with poor prognosis [169, 170, 171]. Xu's experiment revealed that SRF promotes EMT and increases tumor migration and invasion in OSCC cells by activating the IDO1 gene promoter and regulating the IDO1/Kyn‐AhR signaling pathway [69]. This effect can be blocked by IDO1 and AhR inhibitors. Similarly, in bladder cancer, IDO1 expression is related to cell migration ability, and silencing IDO1 reduces EMT through the IL‐6/STAT3/PD‐L1 pathway [9]. MMPs, a family of zinc‐dependent metalloproteinases, degrade ECM components, facilitating tumor cell migration to nearby tissues. In Lewis lung carcinoma cells [70], IDO1 overexpression upregulates MMP‐2 and MMP‐9 via the JAK2/STAT3 pathway. COL12A1 encodes a collagen protein that interacts with collagen types I and III to form homotrimers [51, 172, 173], activating the Collagen I/integrin β1 pathway [174]. COL12A1 is strongly associated with migration, invasion, and metastasis in breast and colorectal cancer. Xiang's study confirmed a positive regulatory loop between IDO1 and COL12A1, both promoting gastric cancer metastasis via the MAPK pathway [51, 175].

TDO2 serves as the main enzyme metabolizing tryptophan in cancer‐associated fibroblasts (CAFs), with the produced kynurenine inducing EMT and cancer cell migration via the AKT/WNK1 axis [176]. In glioma, TDO2 is upregulated and plays a decisive role in tryptophan metabolism, and its expression is related to glioma cell motility [45]. Anoikis is a type of apoptosis caused by the loss of cell attachment to the extracellular matrix. In triple‐negative breast cancer (TNBC) [156], TDO2 expression is upregulated and strengthens anoikis resistance and metastasis through the TDO2‐AhR signaling axis. These results indicate the important role of TDO in tumor migration.

Interestingly, recent studies have found that KYNA inhibits cell migration in glioblastoma [135] and renal cell carcinoma [137], but the specific mechanisms are still under investigation. Previous studies have found that nicotine activates α7nAChR, leading to ERK pathway activation, promoting cell proliferation and neovascularization. Tu et al. also reported the key role of this receptor in gastric cancer progression and metastasis [177]. As an α7nAChR antagonist, Kyna may act through this pathway. We previously discussed the tumor specificity of KYUN in tumor immunity. In gastric cancer, GPx2 knockdown‐induced ROS accumulation promotes KYNU expression, which subsequently degrades Kyn, thereby inhibiting AhR‐mediated tumor progression and EMT [178].

Recently, a new tryptophan metabolism‐related enzyme, interleukin‐4‐induced‐1 (IL4I1), was discovered [8]. IL4I1 converts tryptophan into I3P, which can further metabolize to produce KynA and I3A. All three metabolites activate AhR, enhancing tumor cell motility and invasiveness. Additionally, IL4I1 is an AHR target gene, suggesting that it can amplify its own expression through positive feedback. Unlike IDO1 and TDO2, IL4I1 is a secretory protein [179]. Sadik et al. found that IL4I1 levels increased in the serum of CLL mice, indicating that IL4I1 could extend AhR activation effects from a local to a systemic level, better facilitating the creation of a supportive environment for tumor growth in metastatic sites [8].

### 2.5 The KYN Metabolites 3‐Hydroxykynurenine (3‐HK) and Hydroxyanthranilic Acid (HAA) Effectively Block Tumor Ferroptosis

Ferroptosis is a unique cell death mechanism characterized by the accumulation of intracellular iron ions, leading to lipid peroxidation, ultimately causing membrane rupture and cell death. Oncogenes and associated signaling pathways mediating ferroptosis resistance facilitate tumor development, survival, and metastasis. Alessandra's research indicates that tryptophan, through the kynurenine pathway, plays a key role in tumor resistance to ferroptosis [71].

1. Tumor cells upregulate IDO1, KYUN, leading to KYN metabolism into 3HK and HAA. Studies revealed that these two compounds maintain HeLa cell viability under ferroptosis inducers (erastin and RSL3) for 48 h [180]. This was also replicated in SKOV3 (ovarian cancer cells), HT1080 (human fibrosarcoma cells), and PT45 (aggressive pancreatic cancer epithelial cells), linking their effects to the inhibition of intracellular ROS and lipid peroxidation [71]. Additionally, Liu found that 3‐HA is the most effective ferroptosis inhibitor in the kynurenine pathway, with KYNU playing a critical role in regulating 3‐HA levels and tumor cell resistance to ferroptosis [180].
  As a 3‐HA metabolic enzyme, HAAO inhibits tumor cell resistance to ferroptosis by depleting intracellular 3‐HA. Gene expression analysis via the TIMER database showed that HAAO has low expression in various cancers, including breast and liver cancer, allowing for the accumulation of 3‐HA in the tumor microenvironment [180]. HAAO expression is negatively correlated with anti‐ferroptosis genes such as SLC7A11, SLC3A2, and ACSL3, and high HAAO expression is associated with improved prognosis in cancers such as breast, kidney, and liver cancer. These data suggest HAAO's potential tumor‐suppressing role via ferroptosis, offering insights for new drug development [180].
2. IDO triggers TRP depletion in the tumor environment, activating the integrated stress response (ISR), which detects amino acid fluctuations and phosphorylates eIF2α, resulting in the upregulation of transcription factors such as ATF4 and NRF2. ATF4 and NRF2, in turn, promote SLC7A5 and SLC7A11 expression, enabling KYN transport into IDO1‐negative cells for further metabolism, expanding the impact. SLC7A11 also serves as a cysteine transporter, and when KYN competitively binds to it, it creates a pseudo‐cysteine deprivation, intensifying ISR activation. Furthermore, ISR activation suppresses mTOR, pausing cell growth and protein synthesis, and redirecting more available cysteine for glutathione production, alleviating lipid peroxidation [71]. It is noteworthy that Liu's research suggests that 3‐HA, as a metabolite of tryptophan, can suppress ferroptosis independently of the NRF2‐SLC7A11 pathway [180].

### 2.6 Thoughts on the Efficacy of IDO1 Inhibitors

It is widely recognized that IDO1, within the KP, suppresses tumor immunity by generating KYN, and inhibitors developed based on this mechanism, such as epacadostat and navoximod, have entered clinical trials. Nevertheless, in combined treatments with IDO1 inhibitors, notable clinical benefits seem absent. As TDO2 has also been shown to promote tumor growth in some cancers, it is reasonable to speculate that TDO2 and IDO2 may act as compensatory pathways to continue KYN production and influence tumor immunity when IDO1 is inhibited. In the development of new drugs, in addition to the non‐enzymatic roles of IDO1 mentioned above, the broad inhibition of IDO1/IDO2/TDO2 should also be considered. AFMID, as a metabolizing enzyme for *N*‐formylkynurenine (NFk), is also highly expressed in tumors. Due to its unique role in KYN production, AFMID inhibitors similarly hold promising potential. Additionally, as an AhR antagonist, Trp can regulate AhR activity by supplementation under appropriate conditions, thereby exerting protective effects. Trp supplementation can be used in combination with broad inhibitors of IDO1/IDO2/TDO2. This combination can ensure the blockage of AhR agonist production, thereby reducing excessive AhR activation.

Standard chemotherapeutic drugs may promote immune evasion by activating the KP; in HNSCC cells, cetuximab induces IDO1, and in melanoma, the anti‐PD1 monoclonal antibody (mAb) nivolumab can induce IL4I1 and IDO1, with nivolumab treatment linked to AhR activation. IL4I1 can increase PD1 expression through the AhR pathway. Although the specificity of many enzymes and their metabolites in the KP across different cancers has not been fully studied, this information suggests that some enzymes in the KP may be affected by current standard treatments, potentially leading to post‐treatment resistance. In future treatment regimens, it will be necessary to screen patients in whom the tryptophan metabolism pathway or IL4I1 contributes to tumor progression to better devise blocking strategies.

## 3 The Function of Indole Pathway in Cancer

The gut microbiota, which has the highest density and diversity among human microbiomes, has been extensively studied in relation to various cancers [181]. It has been found that 20% of human malignancies are associated with dysbiosis of the human microbiota [182]. Previous studies have confirmed that gastrointestinal microbial metabolites such as fatty acids (SCFAs), microbial tryptophan catabolites (MTCs), and bile acids (BAs) have a significant impact on tumor development [183, 184]. These metabolites are also linked to the initiation and progression of colorectal inflammation and tumorigenesis [185, 186, 187]. Increasing evidence suggests that the gut microbiota influences tumors and tumor drug efficacy by regulating tryptophan metabolism.

### 3.1 The Indole Pathway Maintains Gut Homeostasis

Previous studies demonstrated that chronic, low‐grade inflammatory diseases (such as inflammatory bowel disease) create a tumor‐promoting environment, leading to colon cancer [188]. Additionally, in cases of damage due to disease, infection, or food, the gut microbiota initiates an adaptive immune response, producing tumor‐promoting factors like IL‐17 and IL‐23, which accelerate tumor growth. Inflammation that occurs when the intestinal barrier is damaged alters the gut microbiota, and the “reselected microbiota” has defects in repairing the intestinal barrier and controlling inflammation, leading to a favorable environment for cancer development. Changes in the gut microbiota subsequently affect the metabolism of SCFAs, MTCs, and BAs [184, 189]. Studies of gut microbiome metabolites have found that colorectal cancer patients have lower levels of indole in their fecal samples compared to healthy individuals, with increased KYN and KYN/Trp ratios in their feces [185]. Moreover, the fecal KYN/Trp ratio positively correlates with tissue IDO1. Restoring indole and its derivatives helps control cancer by protecting the intestinal barrier and reducing inflammation, thereby maintaining gut homeostasis.

#### 3.1.1 Protecting the Intestinal Barrier

In the intestine, the Pregnane X Receptor (PXR) is a crucial regulator of innate immune responses and responds to xenobiotic injury [190]. Venkatesh's experiments showed that PXR in intestinal epithelial cells is vital for maintaining intestinal barrier integrity and regulating intestinal inflammation [191]. The loss of PXR leads to disrupted tight junctions between intestinal epithelial cells, increasing intestinal permeability. Tryptophan metabolites, such as indole‐3‐propionic acid (IPA) and indole acetic acid (IA), protect the intestinal barrier through PXR. In patients with ulcerative colitis, IPA levels are reduced, but dietary enhancement of IPA increases the expression of tight junction proteins, IL‐22, and AhR, and helps restore the balance of the gut microbiome [192, 193]. In a DSS (dextran sulfate sodium)‐induced colitis mouse model, IPA promotes Muc2 gene expression through PXR, enhancing mucin secretion [20]. Tight junction proteins reduce intestinal permeability, and IL‐22 supports mucosal immunity and integrity by inducing epithelial cells to produce antimicrobial peptides and mucins [194]. AhR also plays an important role in gut homeostasis (as explained below). In cancer associated with colitis, Toll‐like receptor 4 (TLR4) acts as a key regulator of intestinal barrier function and inflammation. By regulating the expression of genes related to intestinal barrier function, TLR4 increases intestinal permeability, worsening the inflammatory environment. IPA binds to PXR and inhibits TLR4, thus protecting the intestinal mucosa [195].

Activation of AhR in intestinal epithelial cells (IECs) is also crucial for maintaining the integrity and function of the intestinal barrier [196]. In Wlodarska's colitis mouse model, commensal bacteria, particularly *Peptostreptococcus russellii* ( *P. russellii* ), activate AhR through the metabolite indole acetic acid (IA), promoting Muc2 gene expression, enhancing Goblet cell differentiation and function, and supporting both gut barrier function and inflammation suppression [20]. In intestinal stem cells, RNF43 and ZNRF3 serve as negative feedback regulators of the Wnt signaling pathway, suppressing excessive stem cell proliferation by modulating the ubiquitination and degradation of Frizzled receptors [197]. When the intestinal barrier is damaged, the lack of AhR downregulates tumor suppressor genes Znrf3 and Rnf43 [197], resulting in abnormal activation of the Wnt‐β‐catenin signaling pathway, which disrupts epithelial cell differentiation from crypt stem cells and worsens gut barrier repair [196]. The indole derivative indole‐3‐carbinole (I3C), metabolized from glucobrassicin in cruciferous vegetables by gut microbiota [196], restores regulation of the Wnt‐β‐catenin pathway, controls epithelial cell differentiation, and reduces inflammation‐induced tumor formation. Additionally, I3C promotes the differentiation of ILC3 cells, which produce IL‐22, thereby maintaining mucosal immunity and integrity [198].

Besides IPA, IA, and I3C, indole‐3‐aldehyde and indole‐3‐acetic acid also affect intestinal barrier integrity and immune cells in mice by activating PXR and AhR [21, 191, 199].

#### 3.1.2 The Indole Pathway Regulates Gut Inflammation

In colitis‐associated colorectal cancer (CRC), AhR not only exerts anti‐inflammatory protective effects by protecting the intestinal mucosa but also suppresses inflammation by promoting the secretion of anti‐inflammatory cytokines [200]. Exposure to physiological concentrations of indole and its metabolites leads to increased expression of anti‐inflammatory cytokines IL‐10 and IL‐22 [20, 201, 202]. IL‐10 secretion further maintains mucin production by goblet cells, while IA activates the NRF2 (Nuclear Factor Erythroid 2‐related Factor 2) antioxidant pathway, promoting antioxidant gene expression and reducing oxidative stress associated with intestinal inflammation [203]. In an inflammation‐related colorectal cancer model, interruption of Trp‐indole‐AhR signaling resulted in significantly elevated mRNA levels of TNF‐α, IL‐1β, and IL‐6 [185]. IPA reduced the expression of TNF‐α, IFN‐γ, and IL‐1β through AhR, lowering the severity of intestinal inflammation in mice [192]. In rheumatoid arthritis [204], intervention to improve gut microbiota and elevate tryptophan metabolites (IA, IAA, IPA) activates AhR, reducing pro‐inflammatory Th17 cells and increasing anti‐inflammatory Treg cells, thereby regulating the Th17/Treg cell balance. Busbee PB et al. found that indole‐3‐carbinol (I3C) activates AhR in colitis mice, reducing pro‐inflammatory Th17 cells associated with colitis and increasing Treg cells involved in maintaining gut homeostasis, thus alleviating inflammation [199]. During inflammation, polymorphonuclear leukocytes (PMNs) are recruited, and their myeloperoxidase (MPO) defends against invading microorganisms and maintains epithelial barrier function by producing hypochlorous acid (HOCl) [205]. However, MPO itself induces PMN degranulation, releasing MPO into the external environment, damaging tissues, and inducing inflammation [206]. Experiments found that indole and its derivatives inhibit MPO by directly binding to its active site or competing with MPO substrates (e.g., H_2_O_2_ and Cl^−^), interrupting the MPO‐driven inflammatory feedback loop, thereby reducing inflammation and promoting tissue repair [207].

**FIGURE 6:**
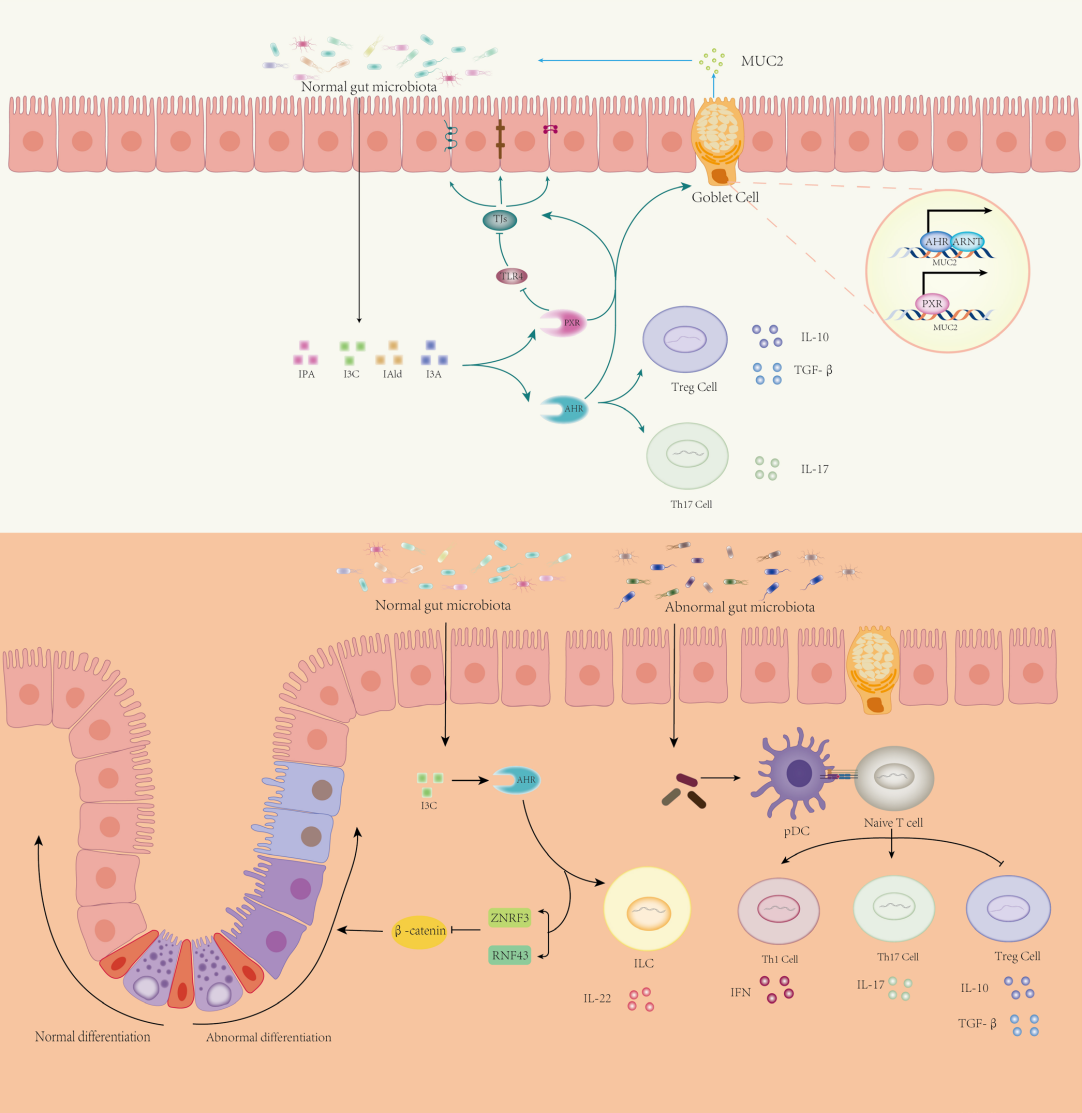
Impact of tryptophan metabolism alterations induced by changes in gut microbiota on gut homeostasis. AHR, aryl hydrocarbon receptor; IFN, interferon; IL‐10, interleukin 10; IL‐17, interleukin 17; IL‐22, interleukin 22; IL‐23, interleukin 23; ILC, innate lymphoid cell; MUC2, mucin 2; Naive T Cell, naive T cell; pDC, plasmacytoid dendritic cell; RNF43, ring finger protein 43; RORγt, retinoid‐related orphan receptor gamma t; TGF‐β, transforming growth factor‐beta; Th1 Cell, T helper 1 cell; Th17, T helper 17 cell; TJs, tight junctions; Treg Cell, regulatory T cell; ZNRF3, zinc and ring finger 3; β‐catenin, beta‐catenin.

In addition to protecting the intestinal barrier and alleviating gut inflammation, studies have found that the indole metabolite IPA reshapes the gut microbiota and maintains gut homeostasis through AhR activation. Previous studies [208] found that IPA inhibits the biosynthesis of tryptophan in *Mycobacterium tuberculosis* and *non‐tuberculous mycobacteria* by binding to TrpE, the first key enzyme in tryptophan synthesis. Additionally, some commensal bacteria that play a protective role in colitis utilize intestinal mucins as a carbon source [20]. In patients with inflammatory bowel disease (IBD), the intestinal mucus layer becomes thinner, affecting the structure and function of the microbiota. As mentioned above, IPA may restore the structure and function of these microbiota by promoting mucin expression (Figure 6).

### 3.2 The Indole Pathway's Impact on Tumor Immune Infiltration

Previous studies have found that indole and its derivatives affect tumor immune infiltration. Sári et al. found that in breast cancer patients, *Escherichia coli* TnaA expression in feces correlates positively with the number of tumor‐infiltrating lymphocytes [209]. Supplementing IPA induced antitumor immune responses, mesenchymal‐to‐epithelial transition, and oxidative stress. Fong discovered that in CRC tumors, the metabolite indole‐3‐carboxylic acid (ICA) from *Lactobacillus gallinarum* competes with Kyn for binding to the aryl hydrocarbon receptor (AhR), counteracting Kyn's interaction with AhR on CD4+ T cells and inhibiting Treg differentiation in vitro [210]. Zhang's experiments showed that the metabolite indole‐3‐lactic acid (ILA) from *Lactobacillus plantarum* L168 can alleviate gut dysbiosis in colorectal cancer mouse models and suppress tumor growth [211]. The suppression of tumor growth by indole‐3‐lactic acid (ILA) is primarily linked to epigenetic mechanisms. First, it promotes the production of IL‐12a in dendritic cells by enhancing the binding of H3K27ac to the IL‐12a enhancer region, thereby promoting the antitumor immune function of CD8+ T cells. Additionally, indole‐3‐lactic acid reduces chromatin accessibility and decreases Saa3 expression in CD8+ T cells, enhancing the function of tumor‐infiltrating CD8+ T cells. Interestingly, indole functions differently in various environments. Hezaveh's experiments found that in pancreatic ductal adenocarcinoma (PDAC), removing dietary tryptophan reduces AhR activity in tumor‐associated macrophages (TAMs) and promotes the accumulation of TNF‐α+IFN‐γ+CD8+ T cells in the tumor [44]. This effect can be blocked by indole supplementation, as TAMs' AhR activity depends on the metabolism of dietary tryptophan into indole by *Lactobacillus* rather than endogenous tryptophan metabolism.

The opposing roles of indole and its derivatives in intestinal and pancreatic tumors are understandable. In the colon, indole derivatives stabilize gut homeostasis by exerting anti‐inflammatory effects and protecting the intestinal barrier, preventing a tumor‐promoting environment. However, in pancreatic tumors, gut microbiota‐derived indole activates macrophages in the pancreas, and AhR activation suppresses antitumor immunity, leading to tumor‐promoting features. Indole and its derivatives, as metabolites of tryptophan, play different roles in various environments, and the role of AhR activation in tumor progression is ligand‐specific. Therefore, it is necessary to conduct metabolomics and single‐cell sequencing of different tumor types to understand how microbial metabolites affect the tumor microenvironment and how AhR signaling impacts different types of immune cells and host immune responses.

### 3.3 Impact of the Indole Pathway on the Kynurenine Pathway and Tumorigenesis

As a tryptophan metabolic pathway, the indole pathway impacts the KP. Sun found that in colorectal cancer patients, changes in gut microbiota decreased fecal indole, raised Kyn and Kyn/Trp ratios, and the Kyn/Trp ratio was positively correlated with IDO1 expression in tissues [185]. Changes in the host gut microbiota reduce the amount of tryptophan metabolized via the microbial indole pathway, decreasing the availability of indole, which has a protective role in the host, and exacerbating the host's inflammatory response [212]. Furthermore, the downregulation of the Trp‐indole metabolic pathway allows more Trp to be metabolized through the KP. The worsening of inflammation can further increase IDO1 activity, leading to effector T‐cell apoptosis via the Kyn pathway and impairing antitumor immunity.

The *Lactobacillus gallinarum* ‐derived metabolite ICA, in addition to competitively binding to AhR with Kyn to inhibit CD4+ T‐cell function and block Treg differentiation, also suppresses IDO1 expression, thus lowering Kyn production in tumors [210]. In renal cell carcinoma (RCC) patients, gut microbiota‐derived tryptophan metabolites are significantly associated with AhR and E‐cadherin expression in RCC. These results suggest that the Kyn metabolic pathway of the gut microbiota may be involved in the pathogenesis of RCC [213].

### 3.4 Impact of the Indole Pathway on Anticancer Drug Efficacy

Pancreatic cancer, lung cancer, breast cancer, and other cancers initially considered sterile have, with further research, been found to also contain microorganisms as part of the tumor tissue itself [214]. In human cancers, intratumoral bacteria mainly reside in the cytoplasm of immune cells and tumor cells [215]. Whether alterations in the gut microbiota or tumor formation occur first remains to be explored. Previous studies indicate that gut microbiota influence tumors; for example, most bacterial communities found in tumor environments also exist in the gut microbiome [216]. In human PDAC patients, the gut microbiota can colonize pancreatic tumors, altering the tumor microbiome, and long‐term survivors have more diverse tumor microbiomes that can induce antitumor responses and the immune system [217].

In addition to affecting tumors, the gut microbiota also influences the efficacy of anticancer drugs. Gajewski's research shows that gut microbiota balance in mice determines responsiveness to certain immunotherapies [218]. Later studies confirmed that gut microbiota composition can improve immunotherapy outcomes (e.g., anti‐CTLA4, anti‐PD‐L1) by modulating the immune system [219, 220, 221]. Gut microbiota is becoming crucial in determining responses to immune checkpoint blockade in cancer therapy, with the indole pathway being one of the mechanisms. In melanoma, the probiotic *Lactobacillus reuteri* releases I3A, activating AhR in CD8+ T cells to produce IFN‐γ, enhancing immune checkpoint inhibitor efficacy and prolonging progression‐free survival (PFS) and overall survival (OS) [222]. In colorectal cancer, *Lactobacillus gallinarum* ‐derived ICA enhances anti‐PD‐1 therapy by regulating the IDO1/Kyn/AhR axis [210].

Indole metabolism influences chemotherapy as well. In a study by Tintelnot J et al. on mPDAC patients, those who responded to chemotherapy had higher levels of 3‐IAA and increased numbers of *Bacteroides fragilis* and *Bacteroides thetaiotaomicron* . Microbiota‐derived 3‐IAA depends on myeloperoxidase (MPO) to enhance the efficacy of the chemotherapy agent FIRINOX in mPDAC treatment [223].

Promoting the growth of tryptophan‐metabolizing bacteria such as *Lactobacillus* or increasing indole production while reducing the utilization of tryptophan by host cancer cells represents a promising new direction for inhibiting tumor growth. However, many challenges remain, such as more accurately identifying microbiota that promote immunotherapy and determining whether bacterial species function uniformly across different tumors. Not all microbiota, such as *Lactobacillus reuteri* , may need to be present in melanomas to exert effects. Thus, it is crucial to identify different groups of gut microbiota and control the balance between immunogenicity and tolerance, as well as develop drugs that can tilt this balance.

Stimulating the growth of tryptophan‐metabolizing bacteria like *Lactobacillus*, or increasing indole production while limiting tumor cells' tryptophan usage, holds promise for tumor inhibition. However, challenges include identifying microbiota that benefit immunotherapy, determining if effects are consistent across tumor types, and whether bacteria like *Lactobacillus reuteri* must be present in melanoma to function. Therefore, recognizing gut microbiota groups, balancing immune response, and designing drugs to shift symbiotic balance are key future tasks.

## 4 Conclusion

Tryptophan metabolism, as a feature of tumor metabolism, is crucial for immune suppression. This article reviews and summarizes the roles of the kynurenine (Kyn) pathway and indole pathway in tumor tryptophan metabolism, with the growth of research over the past decade providing us with a deeper understanding. By utilizing the mechanistic characteristics of tumor tryptophan metabolism, we developed related drugs, which showed significant efficacy in early clinical trials. Although Epacadostat failed to demonstrate additional benefits when combined with pembrolizumab in a randomized phase III study for metastatic melanoma, a review of past research suggests several key factors that may have contributed to the treatment failure, including the compensatory activation of TDO following IDO1 blockade by Epacadostat and the non‐enzymatic roles of IDO1. This provides insights for improving therapeutic strategies. The emergence of IDO1 inhibitors and the sensitizing effect of gut microbiota on immunotherapy present new directions for cancer treatment. More detailed research is needed to understand the specific roles of related metabolites with AhR and to accurately identify gut microbiota that are beneficial to immunotherapy.

## Author Contributions

**Zhanhui Lu:** conceptualization, data curation, writing – original draft, validation, software. **Chengcheng Zhang:** writing – original draft, writing – review and editing, data curation, project administration, supervision. **Jia Zhang:** writing – review and editing, writing – original draft, supervision. **Wan Su:** writing – review and editing, funding acquisition, supervision. **Guoying Wang:** methodology, data curation, writing – original draft, writing – review and editing, software. **Zhongqi Wang:** writing – original draft, writing – review and editing, project administration, funding acquisition, resources.

## Ethics Statement

The authors have nothing to report.

## Conflicts of Interest

The authors declare no conflicts of interest.

## Data Availability

The authors have nothing to report.
